# Whole-Genome Sequence Accuracy Is Improved by Replication in a Population of Mutagenized Sorghum

**DOI:** 10.1534/g3.117.300301

**Published:** 2018-01-25

**Authors:** Charles Addo-Quaye, Mitch Tuinstra, Nicola Carraro, Clifford Weil, Brian P. Dilkes

**Affiliations:** * Department of Biochemistry, Purdue University, West Lafayette, Indiana 47907; † Department of Agronomy, Purdue University, West Lafayette, Indiana 47907

**Keywords:** accuracy, EMS, mutagenesis, mutants, mutations, polymorphisms, rare variants, SNP, sorghum

## Abstract

The accurate detection of induced mutations is critical for both forward and reverse genetics studies. Experimental chemical mutagenesis induces relatively few single base changes per individual. In a complex eukaryotic genome, false positive detection of mutations can occur at or above this mutagenesis rate. We demonstrate here, using a population of ethyl methanesulfonate (EMS)-treated *Sorghum bicolor* BTx623 individuals, that using replication to detect false positive-induced variants in next-generation sequencing (NGS) data permits higher throughput variant detection with greater accuracy. We used a lower sequence coverage depth (average of 7×) from 586 independently mutagenized individuals and detected 5,399,493 homozygous single nucleotide polymorphisms (SNPs). Of these, 76% originated from only 57,872 genomic positions prone to false positive variant calling. These positions are characterized by high copy number paralogs where the error-prone SNP positions are at copies containing a variant at the SNP position. The ability of short stretches of homology to generate these error-prone positions suggests that incompletely assembled or poorly mapped repeated sequences are one driver of these error-prone positions. Removal of these false positives left 1,275,872 homozygous and 477,531 heterozygous EMS-induced SNPs, which, congruent with the mutagenic mechanism of EMS, were >98% G:C to A:T transitions. Through this analysis, we generated a collection of sequence indexed mutants of sorghum. This collection contains 4035 high-impact homozygous mutations in 3637 genes and 56,514 homozygous missense mutations in 23,227 genes. Each line contains, on average, 2177 annotated homozygous SNPs per genome, including seven likely gene knockouts and 96 missense mutations. The number of mutations in a transcript was linearly correlated with the transcript length and also the G+C count, but not with the GC/AT ratio. Analysis of the detected mutagenized positions identified CG-rich patches, and flanking sequences strongly influenced EMS-induced mutation rates. This method for detecting false positive-induced mutations is generally applicable to any organism, is independent of the choice of *in silico* variant-calling algorithm, and is most valuable when the true mutation rate is likely to be low, such as in laboratory-induced mutations or somatic mutation detection in medicine.

Forward and reverse genetics are ubiquitous methods for gene function discovery (Page and Grossniklaus 2002; Alonso and Ecker 2006; Nordborg and Weigel 2008; Schneeberger and Weigel 2011). The principal step in these methods is the induction of mutations in the germline of an organism using various categories of high energy (such as UV light, X-rays, γ rays, and fast neutrons) or chemical mutagens (such as EMS, MMS, and ENU). The objectives are the production of genetic diversity in a population and the identification of phenotypic variants (Waugh *et al.* 2006). The chemical EMS is extensively used for mutagenesis (Sega 1984), and predominantly induces G:C to A:T transitions in the genome of a treated organism (Westergaard 1957; Loveless 1958, 1969; Krieg 1963; Prakash and Sherman 1973; Coulondre and Miller 1977; Singer *et al.* 1978). The accurate detection of these induced mutations is critical to establish evidence of gene function.

The ascendancy of cost-effective DNA sequencing methods has enhanced our capacity to detect induced polymorphisms (Metzker 2010), and several sequencing-based methods have been proposed for the detection of induced polymorphisms in plant genetic studies (Nordborg and Weigel 2008; Rowan *et al.* 2011; Schneeberger and Weigel 2011; Nordström *et al.* 2013; Schlötterer *et al.* 2014; Schneeberger 2014). Despite successes and widespread use of these methods, their accuracy is impacted negatively by the inherently higher error rates of NGS technologies (Nakamura *et al.* 2011; Nielsen *et al.* 2011), disparities in variant-calling methods (O’Rawe *et al.* 2013; Cheng *et al.* 2014), qualities of *in silico* reconstructed reference genome sequences (Cheung *et al.* 2003), and the genome complexity of target organisms (Estivill *et al.* 2002; Fredman *et al.* 2004; Robasky *et al.* 2014). Improving the accuracy of methods for detecting induced polymorphisms in a cost-effective manner would be valuable for gene function discovery studies outside of genetic model species, which are often characterized by small genome sizes.

Sorghum is a fully-sequenced panicoid grass, an important source of food and feed, and a potential biomass for biofuel production (Paterson 2008). It is well adapted to hot, semiarid conditions, nutrient-poor soils, and has particularly high drought tolerance relative to other cultivated cereals (Paterson *et al.* 2009). At 0.8 Gb, the sorghum genome is more compact than maize (2.3 Gb) and sugarcane (10 Gb), and has not undergone a recent polyploidy event (Arumuganathan and Earle 1991; Peterson *et al.* 2002; Swigoňová *et al.* 2004; Price *et al.* 2005). Thus, it offers a potentially simpler system in which to discover mechanisms of drought tolerance, plant nutrition, weed biology, the evolution of C4 photosynthesis, and undertake biofuel research. Recent forward genetics studies using EMS mutagenesis have provided key insights into the function of genes involved in insect–herbivory defense, cell wall and lignin biosynthesis, protein digestibility, leaf vein density, and plant height (Pedersen *et al.* 2005; Peters *et al.* 2009; Xin *et al.* 2009; Blomstedt *et al.* 2012; Koegel *et al.* 2013; Krothapalli *et al.* 2013; Petti *et al.* 2013, 2015; Wu *et al.* 2013; Sattler *et al.* 2014; Rizal *et al.* 2015; Scully *et al.* 2015; Addo-Quaye *et al.* 2017). The recent characterization of mutations in pooled samples derived from a population of 256 EMS-mutagenized sorghum BTx623 lines is a major step toward improving resources for sorghum genetics (Jiao *et al.* 2016). The availability of such large-scale collections of high-quality and well-annotated mutations will play a pivotal role in addressing key questions in plant science research (Grierson *et al.* 2011; Bevan *et al.* 2017).

We have created an additional EMS-mutagenized sorghum population of ∼12,000 lines, and present here the large-scale detection and analysis of SNPs and insertions/deletions (indels) derived from low-coverage NGS of 586 of these individuals. We treated these individuals as a population of replicates of sequencing error to remove false positive SNP calls. This cost-effective and accurate method effectively detects *bona fide* EMS-induced mutations and eliminates false positive calls. We characterized the predicted impact of these mutations on sorghum gene function and created a database of mutation descriptions, the corresponding altered genes, and the gene function descriptions of their homologs in *Arabidopsis* and maize. This method can be applied generally to genetic studies in most organisms, and is independent of the *in silico* variant-calling method utilized. We anticipate that this valuable genetic resource and analytical approach will be useful to geneticists, plant breeders, and the general plant science community.

## Materials and Methods

### Mutagenesis and DNA preparation

Seeds of the reference sorghum BTx623 genotype grown at Purdue University were treated with EMS (Sigma-Aldrich), by imbibing the seeds overnight in distilled water at 20° with shaking (150 rpm) and then soaking in 1 L of 45 mM EMS in 10 mM KH_2_PO_4_ (pH 7.0) at 20° for 6 hr shaking at 150 rpm. Seeds were then washed 10 times with 1 L of distilled water and allowed to air dry at room temperature prior to planting. M_0_ seeds were planted in an isolation block at ∼20 seeds/m. Unmutagenized BTx623 rows were interspersed with mutagenized seeds to serve as pollen parents to improve seed set. Plants were allowed to open pollinate and one head was harvested from ∼12,000 mutagenized plants, dried, and threshed individually to produce M_1_ seed. A total of 20 M_1_ seeds were then planted for 7304 lines. The 10th plant in each plot was self-fertilized to produce M_2_ seed. A total of 40 M_2_ seeds from 4800 lines were planted to score for segregating phenotypes. M_2_ plants in each plot were self-fertilized to produce M_3_ seed. M_3_ seeds for each line were planted in the greenhouse. For each line, adult leaf tissue was harvested from a single plant for DNA preparation and stored at −20°; the same plant was then self-fertilized by controlled pollination to produce M_4_ seed. All DNA was prepared using a CTAB extraction as described by Xin and Chen (2012), except that incubations were done at 55° rather than 60°. The M_4_ seeds were also reserved for stock center deposit, phenotypic assessment, and future research.

### Sequencing protocols

DNA was processed using the Illumina TruSeq DNA PCR-Free LT Library Preparation protocol, to make single indexed, 550 bp insert libraries. These were titred individually using an Applied Biosystems Step One kit with Kapa Biosystems Illumina Library Quantification Kit reagents. After flowcell clustering using a TruSeq PE Cluster Kitv3-cBot-HS kit, sequence data were generated as paired, 101 base reads by an Illumina HiSequation 2500 using SBS v3-HS reagents. “Stage 1” pools of 24 samples with compatible indices were created and a single lane of each pool was sequenced. Based on demultiplexing results from the initial lane of sequence, the library titres were refined and a new “stage 2” pool created to correct for excesses/deficiencies in numbers of reads per library from the first lane. Two lanes of the stage 2 pool sequence were then run. Reads from all three runs were merged for each library.

### NGS read mapping

Illumina HiSeq paired-end sequencing reads were mapped to version 2.1 of the sorghum genome reference sequences (Paterson *et al.* 2009) downloaded from the Phytozome (version 9.1) web portal (Goodstein *et al.* 2012). Read alignment was performed using the BWA program for short reads alignment (Li and Durbin 2009; version 0.6.2) and the variant calls were computed using the SAMtools software suite (Li *et al.* 2009; version 0.1.18). Genome coverage was estimated from the alignments in the BAM files using the *genomeCoverageBed* command in the BEDTools genome arithmetic software suite (Quinlan and Hall 2010; version 2.17.0). Insert sizes for the paired-end reads were estimated using the *CollectInsertSizeMetrics* command in the Picard software package (http://broadinstitute.github.io/picard; version 1.80), with parameter option “VALIDATION_STRINGENCY=LENIENT.”

### Detection of genomic positions with nonreference alignments

Whole-genome variant calling started with the detection of positions with statistical support from nonreference sequence in each sequenced sorghum individual. We used the SAMtools *mpileup* command to compute mapping information for all genomic positions in every BAM file. We selected the following command line parameters to ensure that only DNA bases in sequenced reads with base call qualities of ≥20 were used in variant detection: “-Q 20 -P Illumina -uf.” The *mpileup* output resulted in a Binary Call Format (BCF) file, which was redirected to the BCFtools *view* program. To produce a subset containing only the possible variant positions, we used the BCFtools *view* command with parameter options: “-vcg” to output a variant call (VCF) file limited to likely variants only. This file contains *bona fide* SNP and indel positions, as well as false positive variant calls that satisfied these low-stringency criteria, which accepted any position at which any read returned a nonreference alignment to be a variant position.

### SNP filtering and refinement

The output of the variant-calling stage was refined in multiple steps. We initially used the *varFilter* command (with nondefault parameter option: “-D 100”) in the SAMtools *vcfutils.pl* Perl utility script to remove variants with a low-quality score (<10), low read coverage (read depth < 2), and that were possibly derived from repetitive origins (read depth > 100). We refer to this initially filtered SNPs as the “Repeat-Filtered” SNPs. We then used the SnpSift component (Cingolani *et al.* 2012a) in the SnpEff java package (Cingolani *et al.* 2012b; version 3.1) for further SNP variant filtering. Using the SnpSift *filter* command with query string “(QUAL>=20) & (isHom(GEN[0]) & (isVariant(GEN[0]),” we retained homozygous SNPs with variant call quality of ≥ 20. This set of filtered SNPs was designate as the homozygous “Q20” SNPs. Previous studies have shown that strand-biased SNPs tend to be a source of false positive SNP detections (DePristo *et al.* 2011). The remaining SNPs were further filtered using the SnpSift *filter* command with query string “(((DP4[0]=0) & (DP4[1]=0)) & ((DP4[2]>0)& (DP4[3]> 0)))” to retain SNPs with at least one nonreference read mapping to both the forward and reverse strands of the reference genome, which had no reference reads at the SNP position. This set is referred to as the “Standard Filtered” SNPs. We then used the SnpSift *filter* command with the query string “((REF=“G” & ALT=“A”) | (REF=“C” & ALT=“T”))” to catalog the subset of the standard filtered SNPs that were either G to A, or C to T, substitutions.

To detect heterozygous SNPs, we modified the above procedure for detecting homozygous SNPs. We replaced the SnpSift *filter* query string “(QUAL>=20) & (isHom(GEN[0])) & (isVariant(GEN[0]))” with “(QUAL>=20) & (isHet(GEN[0])) & (isVariant(GEN[0]))” to select the heterozygous Q20 SNPs. The low read depth in our genomic sequencing required a relaxed stringency for the presence of mapped reference reads at the heterozygous SNP genomic position to retain likely heterozygous positions. We accomplished this by replacing the SnpSift *filter* query string “(((DP4[0]=0) & (DP4[1]=0)) & ((DP4[2]>0) & (DP4[3]> 0)))” with “((DP4[2]>0) & (DP4[3]> 0)).” This checks only for the mutant allele by determining the presence of at least a single nonreference read on both the forward and reverse strands.

### Indel detection and refinement

Indels in the VCFs were cataloged using the SnpSift *filter* command with query string “(exists INDEL).” Homozygous and heterozygous indels with variant quality of ≥20 were retained using the *filter* command with query strings “(QUAL>=20) & (isHom(GEN[0])) & (isVariant(GEN[0]))” and “(QUAL>=20) & (isHet(GEN[0])) & (isVariant(GEN[0])),” respectively. To eliminate homozygous and heterozygous indels from positions with strand-biased alignment, we required variant calls on both strands using the *filter* query strings “(((DP4[0]=0) & (DP4[1]=0)) & ((DP4[2]>0) & (DP4[3]> 0)))” and “((DP4[2]>0) & (DP4[3]> 0)),” respectively.

### Detection of probable error-prone genomic positions

Following the set of variant filters described above, the standard filtered SNPs detected in the preliminary set of 570 sequenced individuals were concatenated into a single list. These alleles were sorted by chromosome and genomic position. The number of appearances of each genomic position among the called SNPs was tallied. To the extent that EMS mutagenesis is stochastic and even in its ability to affect mutation, the same SNPs should rarely occur in more than one individual in an organism with a sufficiently large genome size. Thus, positions with a tally of two or more SNP call instances were recorded as probable error-prone positions. We refer to this set of probable error-producing positions in the sorghum reference genome (version 2.1) as the Error-Prone SNP Positions. This list of identified positions was then used to subtract the error-prone portion of the genome from the standard filtered SNP calls for each of the final 586 sequenced individuals, and included the preliminary 570 sequenced individuals. The remaining, unrepeated or sample-specific SNPs were unique to each line. The subtracted repeat SNPs and the unrepeated SNPs we refer to as the Replicate and Nonreplicate SNPs, respectively. We applied the same filtering technique to the indel variant calls using the indel event starting nucleotide position of each indel as the sort item. Indels starting at positions present in more than one line were removed from all indel lists. To detect possible false negative replicate SNPs with origins in mutational hotspots (Krasileva *et al.* 2017), we further grouped the replicate SNP positions by the count of the number of individuals containing the SNP position. For each group, we calculated the percentages for each substitution type and performed signal-to-noise analysis by using the G:C to A:T substitution percentage as the signal. Based on the signal-to-noise analysis, we then created a separate catalog of tentative false negative G:C to A:T SNPs. Lists of these positions, and the counts of observed SNPs and indels in the 586 lines, are provided as supplemental information.

### Classification of SNP and indel effects on gene function

We used the SnpEff program (version 3.1) to predict and classify the effects of the detected SNPs and indels on sorghum gene function. To use SnpEff, we had to create a custom SnpEff database and entry for the sorghum genome reference assembly (version 2.1) by using the SnpEff *build* command with parameters “-gff3 -v.” Predicted effects of the detected SNPs and indels were computed by the SnpEff *eff* command with parameter option “-c.” We retained only the subset of variants predicted to have either medium or high impact on an encoded protein sequence. Finally, the genome-wide distribution of indel lengths was estimated using the vcftools program (version 0.1.14) with parameter option “–hist-indel-len” (Danecek *et al.* 2011).

### Gene function annotation

We used the predicted protein sequences of *Sorghum bicolor* BTx623 (version 2.1), maize B73 (annotation version 5b.60), and *Arabidopsis thaliana* (TAIR release version 10) from Phytozome (version 9.1) to annotate the SNP-encoding genes. We created protein BLAST search databases for the maize and *Arabidopsis* sequences using the *makeblastdb* command in the BLAST suite of programs (Altschul *et al.* 1990; Camacho *et al.* 2009; version 2.2.30+), with the nondefault parameters: “-input_type fasta –dbtype prot.” We aligned each sorghum protein sequence to the maize and *Arabidopsis* protein sequences using the *blastp* (protein BLAST) command with nondefault parameters: “-evalue 1E-05 -num_threads 16 -max_target_seqs 5 -outfmt 6 -seg yes.” The gene function description files for sorghum (version 2.1), maize B73 (version 5b.60), and *Arabidopsis* (TAIR version 10) were obtained from Phytozome, and annotations were appended as additional columns to the VCFs for the affected sorghum locus, along with the gene identifiers and annotations for the best maize hit and best *Arabidopsis* BLAST hit. The top hits for maize and *Arabidopsis* were appended only when BLAST *e*-values were less than a threshold value of E−30.

### Search for sequence motifs in flanking sequences of SNPs

We converted the formatted VCFs containing the nonreplicate and likely EMS-induced homozygous SNPs into the BED format by replacing the VCF genomic position with the respective BED start and end positions of a 21 bp window. The set of flanking sequence contexts of all the SNP positions was saved as both FASTA and TAB delimited formats using the BEDTools *getfasta* program with parameters “-bed -fo” and “-bed -tab,” respectively. A subset of 10,000 sequences was randomly selected with uniform distribution from the FASTA-formatted file containing the 21 nucleotide (nt) sequences (SNP and each flank). Using the sorghum reference genome sequences (version 2.1), we generated a third-order background Markov model using the *fasta-get-markov* utility program in the MEME ungapped motif-finding software suite (Bailey *et al.* 2009; version 4.9.1) with nondefault parameter: “-m 3.” To detect an enrichment of ungapped motifs in the sequence context of the 10,000 selected SNPs, we used the parallel version of the *meme* program for ungapped motif discovery with parameters “-dna -mod zoops -revcomp -bfile BKGRND -oc directory -maxsize 300000 -p 16 -nmotifs 50 -minw 5 -evt 0.01.” Using these parameters, we limited the search results to the top 50 ungapped motifs with minimum length of 5, an *e*-value threshold of 0.01, and the profiling evaluated both the original sequences and their reverse complements.

### Analysis of the DNA sequence context of the entire set of likely EMS-induced mutations

The FASTA-formatted file containing the extracted DNA sequence contexts of all the likely EMS-induced SNPs was partitioned into two files, based on whether the SNP reference base was a G or C nucleotide. For each of the 20 positions surrounding the SNP, we tabulated the number of occurrences for all four DNA nucleotide types in each sequence. The tabulation was performed separately for each of the above two files. Next, we repeated the process using the same sample sizes but with two files containing 21 nt sequence contexts of randomly selected G and C reference base positions in the sorghum genome. For each DNA nucleotide type, and at each of the 20 positions, we computed the percentage changes (deviations) in the number of occurrences from the random data set to the EMS-induced SNP data set. We analyzed the tabulations of the pairs of data sets for the G and C reference base cases separately.

### Detection of paralogs of the sequence contexts of error-prone SNP positions

Due to the complexity of the sorghum genome, we determined that ∼50 flanking bases are sufficient to distinguish the sequence context of a specified genomic position from another. We hence extracted the 51 nt sequence context for the detected error-prone SNP positions. These were aligned to the sorghum reference genome sequences in a BLAST-indexed database using the *blastn* program in the BLAST software suite (version 2.2.30+) with parameter options: “-evalue 1.0E-10 -num_threads 16 -outfmt 0 -ungapped -dust no -word_size 11.” The BLAST *e*-value threshold was selected based on initial preliminary runs in order to exclude a high percentage of spurious matches in the alignment results. The BLAST ungapped alignment output results were initially parsed to retain only a subset of alignment results, which consists of global alignments. The stringency of the filtering was subsequently increased to only retain global alignments containing a single substitution, coinciding with the 26th position (error-prone SNP position). The alignments and the single nucleotide substitution base were then cataloged. For each of the error-prone SNP positions, we compared the DNA base in the single nucleotide substitution in the BLAST alignments with the false positive SNPs calls at the error-prone reference position, and kept track of matching and mismatching bases. We repeated this procedure for two other data sets, with each consisting separately of 58,000 randomly selected genomic positions, and likely EMS-induced homozygous SNPs. We tabulated and compared the BLAST results for the three data sets.

### Annotation of likely adverse effect of missense mutations using SIFT

We followed the recommended steps for the setup and application of the SIFT program to predict the effect of missense mutations on protein function (Kumar *et al.* 2009; version 5.2.2). We downloaded the UniRef90 protein database (UniProt Consortium 2014; Suzek *et al.* 2007; release 2015_03) from The European Bioinformatics Institute website (http://www.ebi.ac.uk/uniprot; retrieved in March, 2015). The FASTA-formatted sequences were then formatted into a protein BLAST database using the *formatdb* command in the BLAST package with parameters: “-i uniref90.fasta -p T –n uniref90.” The SnpEff annotated VCF-formatted files were parsed to extract the subset of likely EMS-induced SNPs, which were predicted to cause missense mutation. The output file consisted of the amino acid changes, their positions in the protein sequences, and the genes that encode them. Using this table, we generated an input file for each sorghum gene transcript with at least one missense EMS-induced SNP. The file for each gene transcript contained all the amino acid changes detected for that gene in all 586 sequenced individuals. The amino acid sequence of each affected gene transcript was downloaded from Phytozome and stored in separate FASTA-formatted files. The individual sorghum protein sequences, and the UniRef90 BLAST database and amino acid changes files for each sorghum gene transcript, were used as input to the *SIFT_for_submitting_fasta_seq.csh* C-shell utility script, which was provided in the SIFT program to predict the effect of missense amino acid changes on protein function. For SIFT prediction, we specified a median conservation value of 2.75 and a SIFT score threshold of 0.05. The *seqs_chosen_via_median_info.csh* script in the SIFT package was modified to increase the CPU time limit from 30 to 60 min (“limit cputime 60m”) and the *psiblast* alignment program was executed in the multithreaded mode (“-num_threads 16”). A mutation was predicted to be deleterious if the computed SIFT score was lower than the threshold. We parsed the output results of the SIFT program to retain amino acid changes with predicted “DELETERIOUS” and “TOLERATED” designations. These designations were appended to the corresponding amino change columns in the EMS-induced SNPs annotation file. The main scripts for the computational pipeline implemented for SNP detection and annotation are included as supplemental information (see Supplemental Material, File S25).

### Data availability

File S1, File S2, File S3, File S4, File S5, File S6, File S7, File S8, File S9, File S10, File S11, File S12, File S13, File S14, File S15, File S16, File S17, File S18, File S19, File S20, File S21, File S22, File S23, File S24, and File S25 are available from the Dryad Digital Repository: https://doi.org/10.5061/dryad.t80gj. The Illumina NGS data sets generated for this project have been deposited in the NCBI SRA (Short Reads Archive), under the Project Title: “Purdue University: Functional Gene Function Discovery Platform for Sorghum,” with the SRA Study Accession Number SRP065118. The BioProject ID# is PRJNA297450 and the BioSample ID#s range from SAMN04128840 to SAMN04129425. Seed samples for the EMS-mutagenized sorghum population used in this study are available on the Germplasm Resources Information Network database.

## Results

### NGS sequencing and read mapping

A total of 29,972,216,082 Illumina NGS paired-end reads were generated from 586 EMS-mutagenized sorghum BTx623 individuals (Table 1). Of these reads, 29,651,940,529 (99%) aligned to the sorghum BTx623 reference genome (Paterson *et al.* 2009). From the alignment, the estimated median paired-end insert size was 527 bp. The average genome coverage of the alignments was 7×. These data were used to identify SNPs that were likely to be the result of the EMS treatment.

**Table 1:** Summary sequencing and alignment statistics for the 586 EMS-mutagenized sorghum BTx623 individuals

	Total	Mean
Reads	29,972,216,082	51,147,126
Mapped reads	29,651,940,529	50,600,581
Properly paired reads	29,288,740,978	49,980,787
Median insert size	—	526
Genome coverage	—	7

### Standard SNP detection and filtering

To detect the high-quality EMS-induced SNPs from the nearly 30 billion sequencing reads, we implemented a highly decoupled SNP prediction and annotation pipeline (See *Materials and Methods*). Initially, we detected 31,178,985 putative SNPs in the 586 EMS-mutagenized individuals. The default criteria for SNP calling resulted in an unreasonably high number of positives: a polymorphism for every ∼14 kb of genome with 53,206 SNP sites per individual (Table 2). The removal of SNPs characterized by low quality, repetitive origin, low read coverage, or strand bias, retained a total of 5,399,493 high-quality standard filtered homozygous SNPs and an average of 9214 per individual (File S1 and Table 2). Similarly, we retained 7,444,547 standard filtered heterozygous SNPs and an average of 12,704 per individual (File S1 and Table 2).

**Table 2:** Summary statistics for the detection of single nucleotide polymorphisms in the 586 EMS-mutagenized sorghum BTx623 individuals

	Homozygous		Heterozygous	
	Total	Mean	Total	Mean
Initial SNPs	13,533,918	23,095	17,645,067	30,111
Repeat filtered SNPs	12,480,017	21,297	15,992,892	27,292
Q20 filtered SNPs	6,906,590	11,786	8,887,909	15,167
Standard filtered SNPs	5,399,493	9,214	7,444,547	12,704
Standard filtered G:C to A:T SNPs only	2,115,636	3,610	2,662,484	4,543
Nonreplicate SNPs	1,275,872	2,177	477,531	815
Nonreplicate G:C to A:T SNPs only	1,247,187	2,128	448,786	766

SNP, single nucleotide polymorphism; Q20, homozygous SNPs with variant call quality of ≥20.

### Prediction of error-prone SNP positions

**Figure 1:**
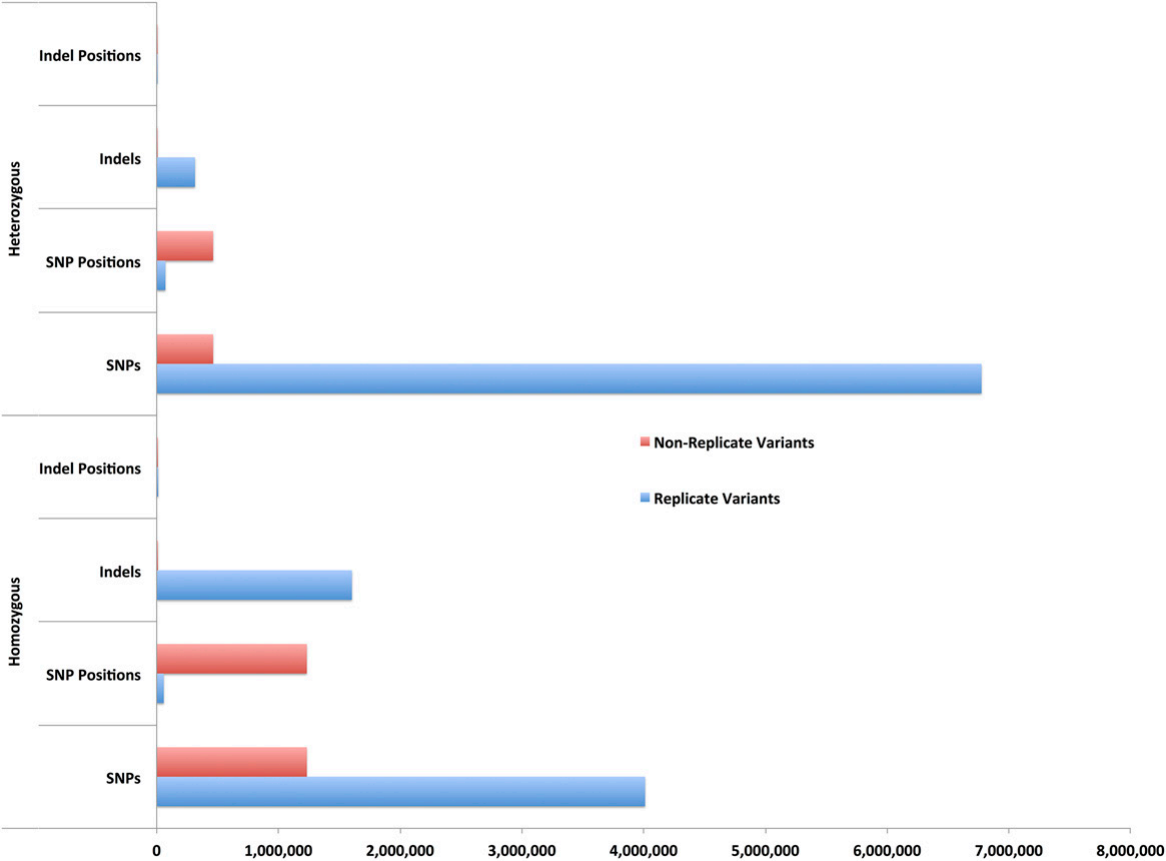
Detection of likely *bona fide* EMS-induced variants by genome origin characterization of the standard filtered DNA variants. In contrast to the nonreplicate or sample-specific variants, the replicate variants were predicted recurrently from relatively few, distinct genomic positions. Over 76% of the standard filtered homozygous SNPs were predicted repeatedly from only 4% of the total distinct homozygous SNP positions in 570 sorghum individuals. For the standard filtered heterozygous SNPs, 94% were predicted repeatedly from only 13% of the distinct heterozygous SNP positions. Similarly, >99% of the homozygous and 98% of the heterozygous standard filtered indels were predicted repeatedly from 56 to 50% of their respective sets of indel positions. EMS, ethyl methanesulfonate; indel, insertion/deletion; SNP, single nucleotide polymorphism.

Because pollen and seed contamination might result in the mistaken resequencing of the same line twice, we compared the SNP calls between lines. This analysis identified 16 lines with >68% overlap between the called SNPs and patterns of SNP coincidence that were consistent with pollen or seed contamination. For additional analysis, we therefore focused on the 570 lines without siblings. For these 570 lines, the SNP detection and standard filtering retained a total of 5,244,176 standard filtered homozygous SNPs produced at 1,290,635 distinct genomic positions. Surprisingly, 4,011,413 of the 5,244,176 SNPs called were predicted repeatedly from only 57,872 distinct genomic positions. This means that roughly 4% of the distinct locations produced >76% of the SNP calls in the sequence analysis of these 570 individuals (Figure 1 and Table S1 in File S26). For example, there were a total of 1085 positions in the sorghum genome, where the same SNP was detected in between 451 and 500 EMS-treated sorghum individuals. This group of SNPs accounted for 515,902 redundant SNP detections (see Table S1 in File S26). The vast majority of these positions are not random poor base calls. For example, for nearly all (57,802 positions) of these replicate SNPs, the predicted change was the same for all lines (File S2). For example, in 567 lines, a C to G substitution was predicted at genomic position 37,580,764 on chromosome 5. This pointed to some structural basis for the repeated observations. Similarly, of the 7,238,377 standard filtered heterozygous SNPs, 6,775,202 SNPs were derived from replicate SNPs at only 71,294 positions. Thus, roughly 13% of the 534,469 distinct genomic positions produced replicate heterozygous SNPs accounting for 94% of the heterozygous SNP predictions in the 570 sorghum individuals (Figure 1).

We observed that only 39% (2,050,166 SNPs) of the standard filtered homozygous SNP predictions were G:C to A:T substitutions (Figure S1 and Table S2 in File S26). Similarly, only 36% (2,588,504 SNPs) of the heterozygous SNPs were G:C to A:T substitutions. With <40% of the retained, high-quality standard filtered SNPs showing signatures of EMS-derived mutagenesis, it is more likely that a significant proportion of the 5,244,176 homozygous and 7,238,377 heterozygous SNPs are false positives. Remarkably, removal of the replicate SNPs showed that, consistent with the experimentally determined effect of EMS mutagenesis, 98% (1,205,140) of the retained nonreplicate or sample-specific homozygous SNPs were G:C to A:T transitions (Figure S2). The second and third largest substitution classes were A:T to G:C transitions and A:T to T:A substitutions, accounting for 0.7% (9066) and 0.5% (6194) of the SNPs, respectively. Similarly, in the case of the heterozygous standard filtered SNPs, after removal of the replicate SNPs, the remaining nonreplicate SNPs were 94% (435,414) G:C to A:T transitions (Figure S2).

**Figure 2:**
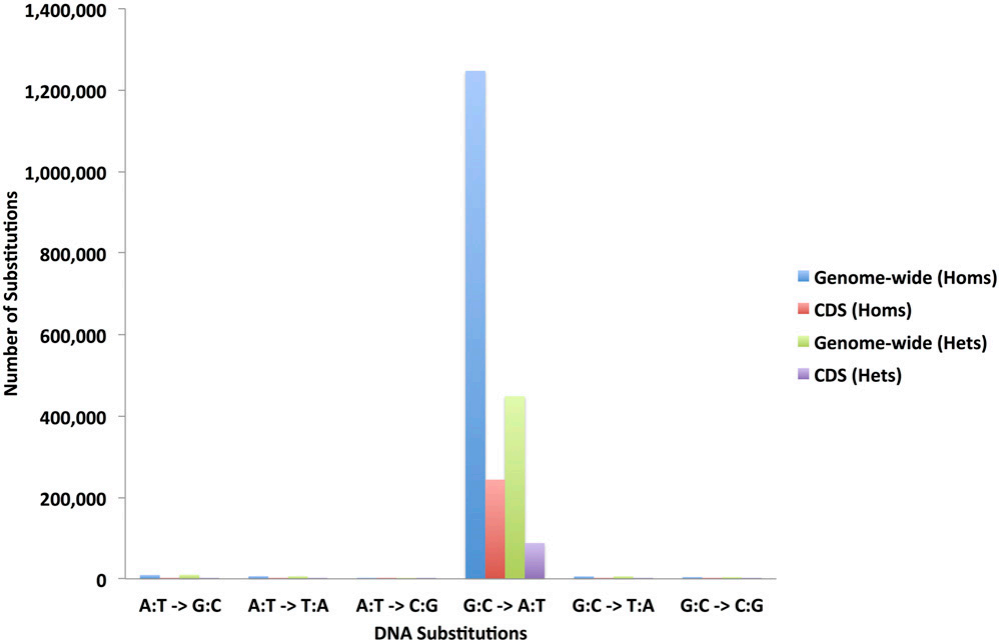
The mutation spectrum and frequency distribution of the likely EMS-induced homozygous and heterozygous mutations in the whole genomes and protein-coding regions of the 586 resequenced sorghum BTx623 individuals. CDS, coding sequence; EMS, ethyl methanesulfonate; Hets, heterozygotes; Homs, homozygotes.

**Figure 3:**
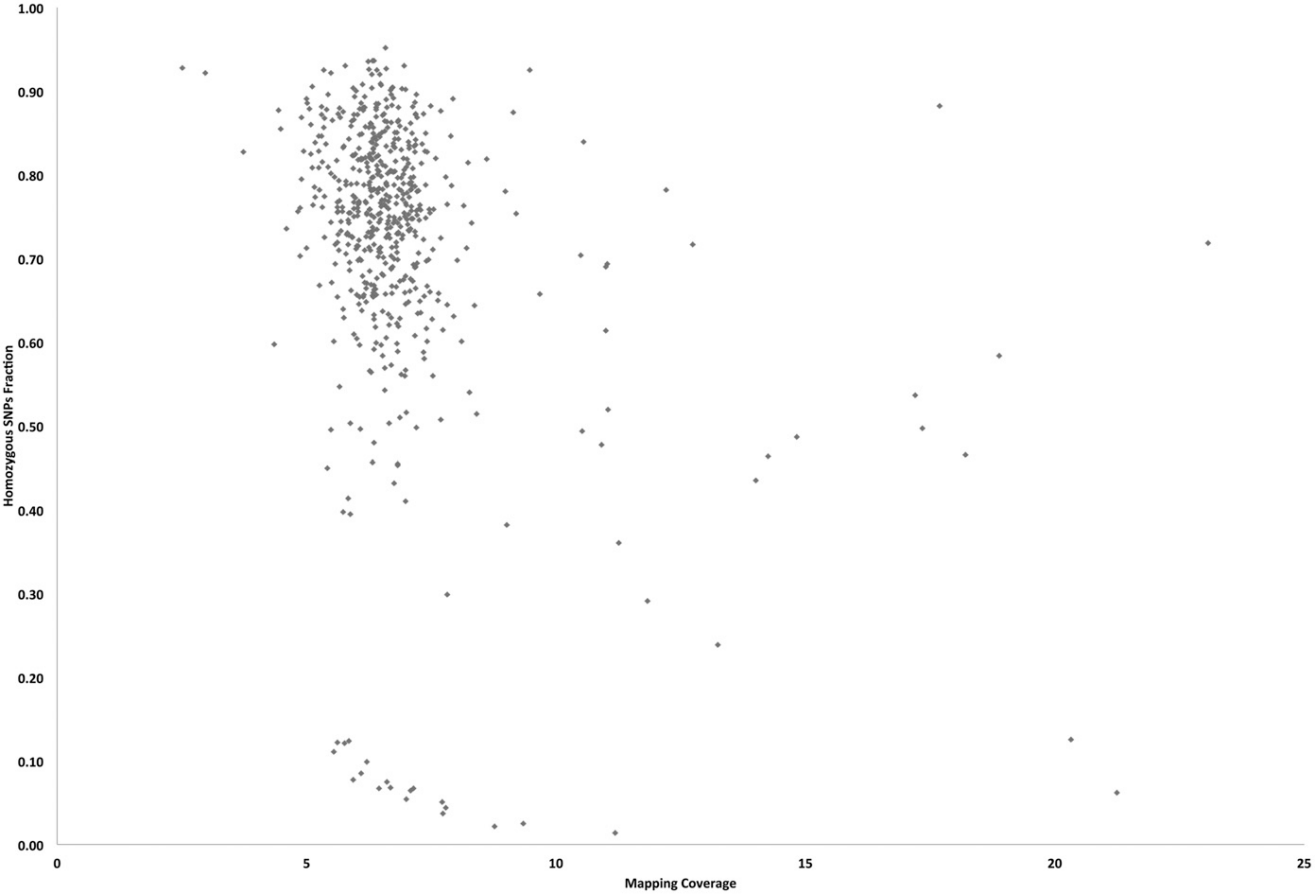
The correlation between sequence mapping coverage and the level of SNPs homozygosity in the genomes of the 586 EMS-treated sorghum BTx623 individuals. EMS, ethyl methanesulfonate; SNP, single nucleotide polymorphism.

The detected probable error-prone SNP positions in the 570 unique lines were used to mask likely false positives from the standard filtered SNPs called for each of the 586 lines. The additional 16 sorghum individuals with overlapping genomic positions were excluded from the masking set, so as not to remove true EMS-induced SNPs that were due to contaminated pedigrees. Cumulatively, 1,275,872 (24%) of the 5,399,493 standard filtered homozygous SNPs were nonreplicate SNPs in the 586 sorghum individuals (File S3 and Table 2), and 98% (1,247,187) were G:C to A:T transitions (Figure 2 and Table S3 in File S26). Protein-coding gene loci comprised 246,062 (19%) of the likely homozygous EMS-induced SNPs and >99% (243,775) were G:C to A:T mutations (Figure 2). Similarly, 477,531 nonreplicate heterozygous SNPs (6%) were retained in the 586 lines after removal of replicates in the 7,444,547 heterozygous standard filtered SNPs (Figure 2, File S4, and Table 2). Hence for our M_3_ population, we observed 27% of the final 1,753,403 nonreplicate SNPs to be heterozygous, and the ratio of homozygous to heterozygous SNPs for the population was 2.7:1. A total of 515 individuals (88%) were homozygous for ≥60% of their SNPs, and only 21 samples were ≤20% homozygous (Figure 3 and File S5). Further analysis revealed a positive correlation (*R* = 0.51) between the final number of predicted heterozygous SNPs and the read mapping coverage for individuals in the mutagenized population. On the other hand, we found a negligible correlation (*R* = 0.07) between the number of homozygous SNPs and mapping coverage. Hence, the final number of heterozygous mutations we predicted may be overestimated, especially for the mutant individuals with lower mapping coverage and lower homozygous percentage. Overall, we predicted an average of 2177 ± 906 homozygous and 815 ± 622 heterozygous nonreplicate SNPs per individual, and observed ∼1 SNP per 243 kb (with one homozygous SNP per 334 kb).

### Detection of likely false negative replicate SNP positions

The signal-to-noise analysis showed a precipitous drop in the G:C to A:T substitution percentages for replicate SNP positions detected in more than two individuals (Table S4 in File S26), and this is further depicted by the point of inflection at *N* = 2 on the *x*-axis of the plot in Figure S3. Over 61% (9291) of the homozygous and 53% (5709) of the heterozygous replicate SNP positions detected in exactly two individuals were G:C to A:T substitutions. Hence, we cataloged an extra 18,582 homozygous and 11,418 heterozygous G:C to A:T substitutions as tentative false negative EMS-induced mutations (File S6 and File S7).

### Functional classification of SNPs and their impacts

**Figure 4:**
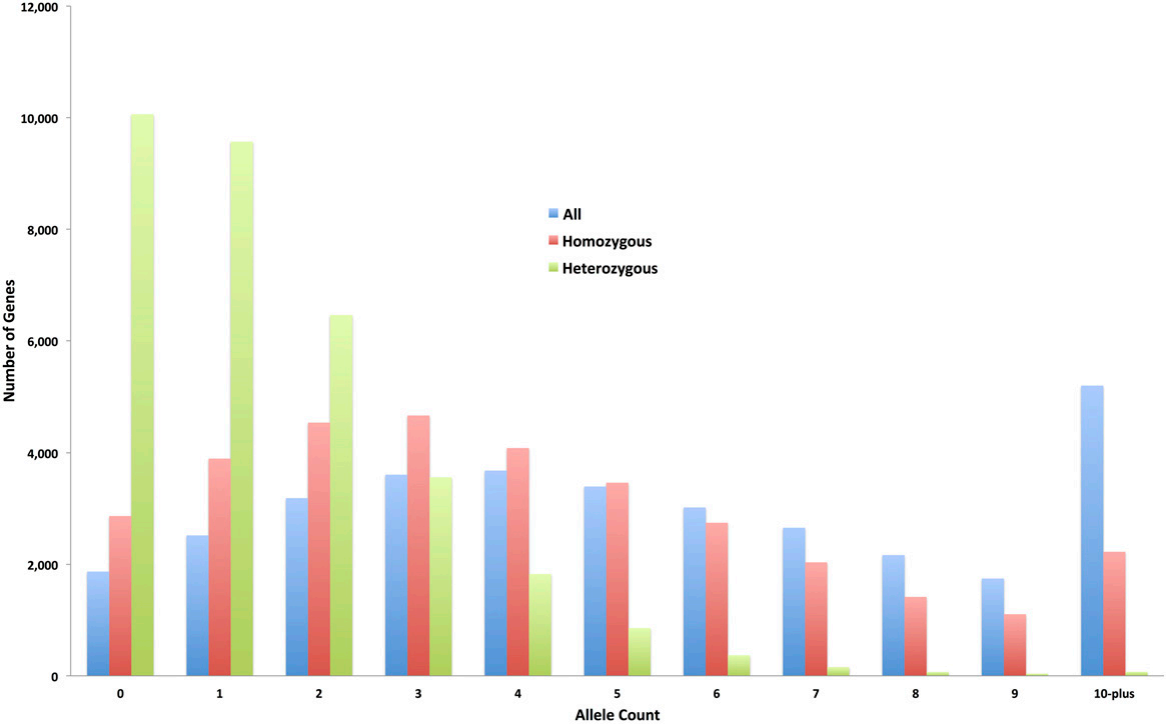
The distribution of allele counts for all genes impacted by likely EMS-induced mutations in the resequenced 586 sorghum BTx623 individuals. Only EMS-induced mutations overlapping protein-coding exons, splice sites, and untranslated regions were considered in the allele counts. EMS, ethyl methanesulfonate.

The majority of the SNP calls were outside predicted protein-coding regions. Intergenic regions and introns contained 1,032,427 (81%) and 109,144 (9%) of the nonreplicate homozygous SNPs, respectively (File S8, File S9, Table 3, and Table S5 in File S26). The remaining 134,301 SNP calls were in protein-coding sequences, with 60,442 effecting changes in protein-coding capacity in 23,866 distinct sorghum genes. Homozygous mutations of high impact (loss of reading frame) were predicted for 4035 SNPs disrupting 3637 distinct genes. These mutations included 2880 nonsense alleles and 1043 mutations removing splice sites. A total of 56,407 nonreplicate homozygous SNPs were predicted to cause nonsynonymous amino acid substitutions in 23,198 distinct sorghum genes. In addition, we detected five lost stop codons associated with nonreplicate SNP changes. These five mutations were not G:C to A:T mutations, as no such mutations can induce a stop codon loss, which may explain the relatively small number relative to the 145 start codon losses. The stop codon losses included a single homozygous and four heterozygous mutations. Synonymous changes from one stop codon to another were associated with G:C to A:T mutations, and we detected 148 synonymous (silent) stop codon substitutions consisting of 114 homozygous and 34 heterozygous mutations. The silent stop-lost mutations included 92 TGA and 56 TAG codon changes to TAA codons (File S10 and Table S6 in File S26). Mutations causing loss of start codons included 111 homozygous and 34 heterozygous changes (Table 3 and Table S5 in File S26). Intergenic regions and introns contained 387,570 (81%) and 42,357 (9%) of the 477,531 nonreplicate heterozygous SNPs, respectively. The heterozygous SNPs also included 1813 high-impact and 19,800 nonsynonymous mutations (File S11, File S12, and Table 3). Taken together, counting mutations in exons, splice sites, and untranslated regions, we detected 188,371 alleles in 31,163 (94%) sorghum genes, including 139,600 homozygous alleles in 30,167 genes (Figure 4). Within protein-coding sequences, we also predicted 79,765 nonsynonymous and 46,234 synonymous mutations, as expected based on codon degeneracy. On average, we predicted seven high-impact (*e.g.*, nonsense mutation or loss of splice site) and 96 amino acid substitution alleles per individual. The average individual also encoded three high-impact and 34 moderate-impact nonreplicate heterozygous SNPs per individual. We estimated an average of 5.7 alleles per sorghum gene. In addition, the tentative false negative SNPs included 40 high-impact and 716 moderate-impact homozygous SNPs, and likewise, 14 high-impact and 326 moderate-impact heterozygous SNPs (File S13 and File S14).

**Table 3:** Classification of the predicted EMS-induced SNPs and their effects in the 586 EMS-mutagenized sorghum BTx623 individuals

SNP Classification	Total		Mean	
	Homozygous	Heterozygous	Homozygous	Heterozygous
Stop gained	2,880	1,363	5	2
Splice site donor	611	304	1	1
Splice site acceptor	432	108	1	0
Start lost	111	34	0	0
Stop lost	1	4	0	0
High impact	4,035	1,813	7	3
Moderate impact	56,407	19,800	96	34
Start gained	3,363	1,237	6	2
5′ UTR	16,308	5,381	28	9
3′ UTR	25,031	9,820	43	17
Introns	109,144	42,357	186	72
Intergenic	1,032,427	387,570	1762	661

SNP, single nucleotide polymorphism; UTR, untranslated region.

### Prediction of indel polymorphisms

We initially detected 3,312,791 indel polymorphisms in the 586 sorghum lines and the standard filtering retained 1,989,078 indels (File S15, File S16, and Tables S7 and S8 in File S26). Less than 1% (17,760) of the remaining indels were retained after replicate indel filtering, indicating an unacceptably high rate of false positives (Figure 1). The average length of the retained indels was 2.6 bp with a remarkably skewed distribution (Figure S4). Among the nonreplicate indels, 77% were ≤ 2 bp in length and 97% of the indels were 10 bases or shorter. A total of 277 and 180 indels were predicted to have high and moderate impact on gene function, respectively. One deletion, in line SbEMS0456-2, disrupts the sorghum ortholog, Sobic.003G386900, of the *Monoculm1* gene from rice, LOC_Os05g42130. The phenotype of this line is reduced height and a complete absence of tillers. Twenty-two indel variations affected splice sites and 1094 occurred in untranslated regions of sorghum protein-coding genes. Another 14,341 and 1947 indels occurred in intergenic and intronic regions, respectively.

### Predicting the impact of missense SNPs on protein function

Over 93% (68,379) of the 73,346 homozygous nonsynonymous EMS-induced SNPs were missense mutations. These affected the coding sequences of 27,943 transcripts encoded by 23,227 distinct sorghum genes. Using the SIFT program in conjunction with the 32,168,296 sequences in the high-quality UniRef90 protein database, we were able to predict the impact of 56,289 missense SNPs. Of these, 25,013 (44%), affecting 12,840 genes, returned a SIFT score <0.05 and likely encode changes deleterious to protein function. The remaining 31,276 SNPs were more likely to be tolerated mutations in 14,559 genes (File S17). Similarly, 24,191 (92%) heterozygous missense mutations were found in 16,118 transcripts encoded by 13,340 (40%) distinct sorghum genes. SIFT analysis predicted the impact of 21,183 of these heterozygous mutations and 9659 (46%) of them were predicted to be deleterious changes. The remaining 11,524 heterozygous SNPs affecting 7417 genes were classified as likely tolerated mutations (File S18). The detailed summary statistics on the sequencing, mapping, SNPs prediction, filtering, annotation, and classification of the homozygous and heterozygous SNPs, as well as the indel polymorphisms, are included as supplemental information (see File S19). The detailed classification of the medium- or high-impact SNPs and indels, and annotations for the affected genes in all 586 sequenced lines, are included as supplemental information (see File S20, File S21, and File S22).

### Analysis of EMS-induced mutations in gene transcripts

**Figure 5:**
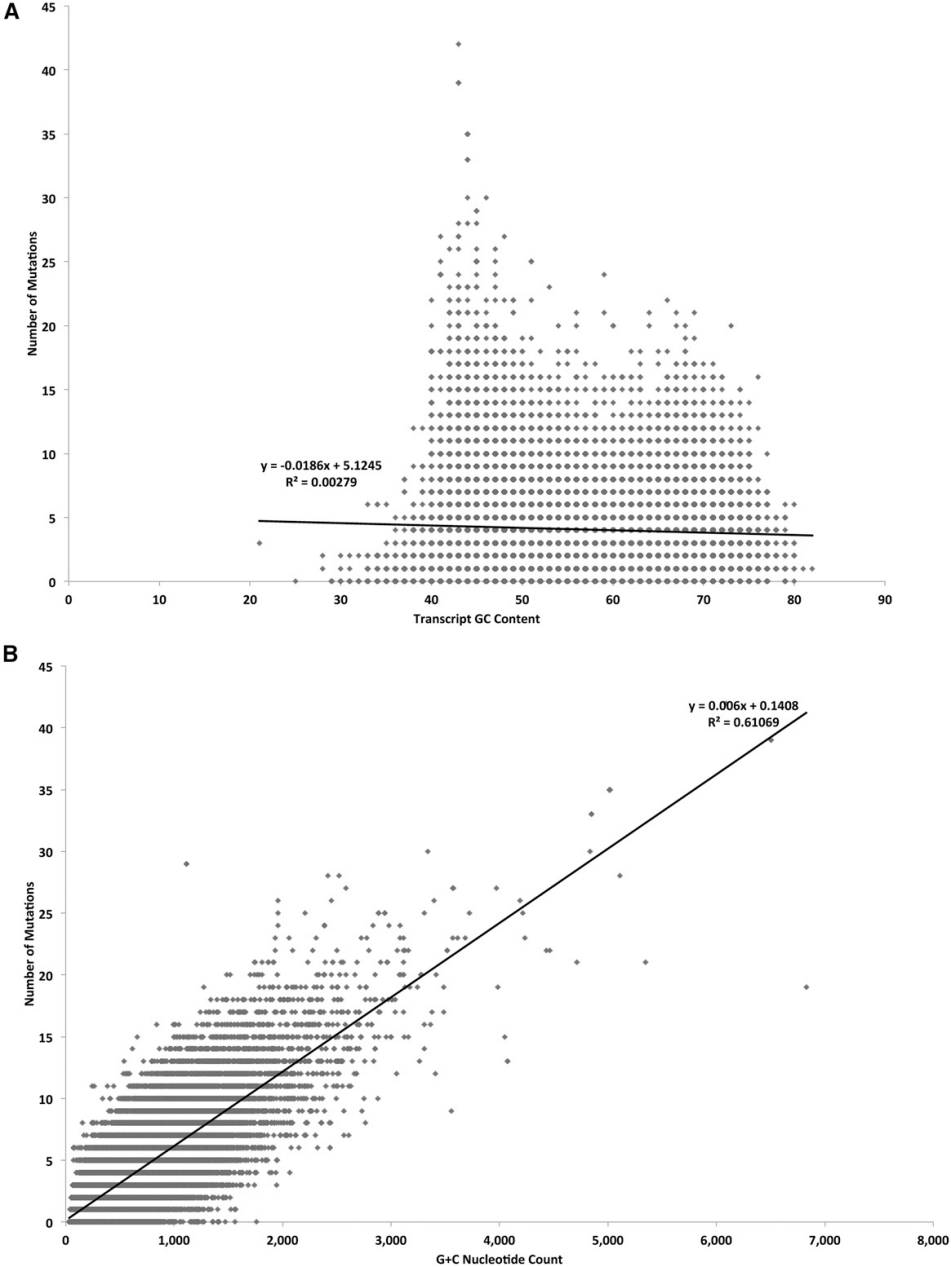

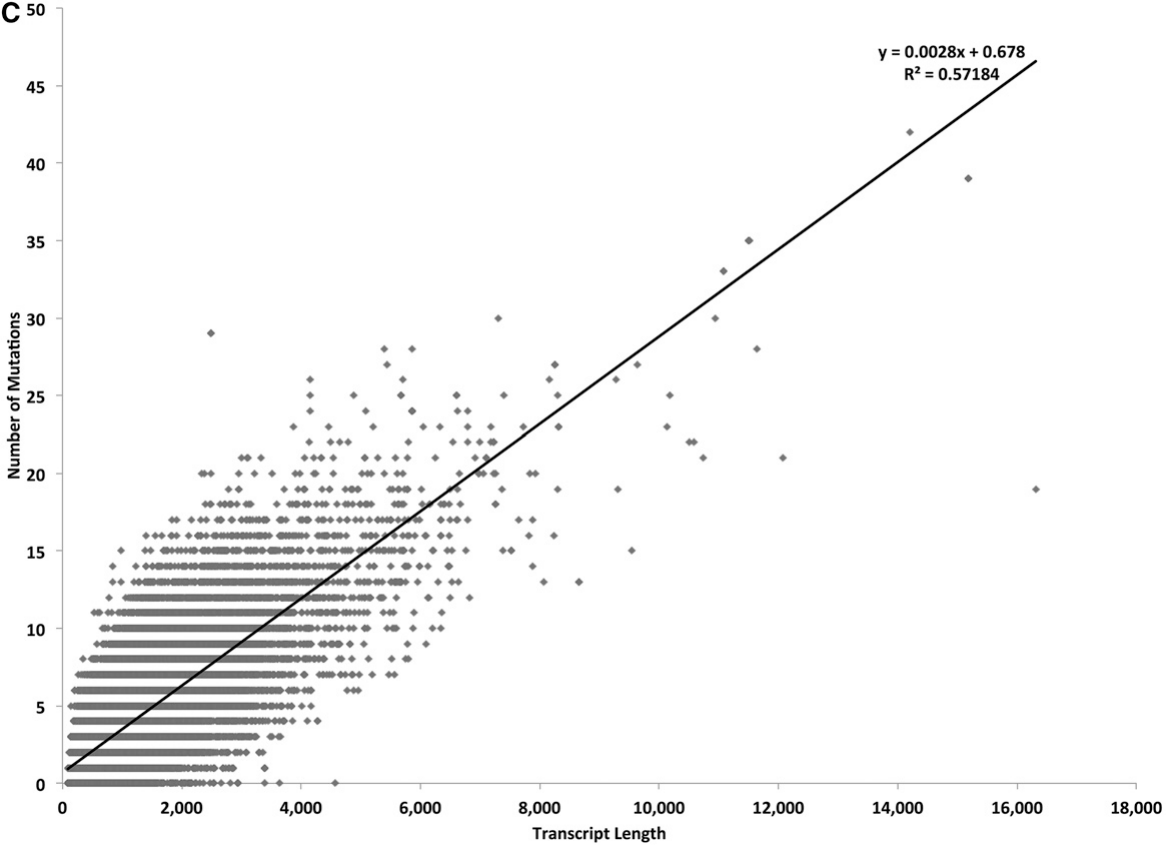
The examination of attributes that impact the number of predicted EMS-induced mutations in a gene transcript. (A) Scatterplot shows no direct linear correlation between mutation count and GC content. (B) Scatterplot depicting a linear correlation between mutation count and G+C count. (C) Linear correlation between mutation count and transcript sequence length.

The sorghum genome annotation includes 39,441 transcripts derived from 33,032 distinct protein-coding genes. We detected SNPs in 89% of these genes (35,221 transcripts and 29,296 genes). Our analysis found no strong transcriptome-wide linear correlation between GC content and the number of SNPs (*r*^2^ = 0.00279; Figure 5A). Unsurprisingly, substituting GC content (dinucleotide percentage calculation) with G+C count, we found that the number of SNPs was linear correlated with G+C count (Figure 5B; *r*^2^ = 0.61069; Figure S5 and Figure S6). Transcripts containing SNPs had mean G and C counts of 361.1 and 340.4, respectively, while the mean count of Gs and Cs for transcripts with no SNPs were 133 and 128.2, respectively, indicating that, in the absence of saturation, genes without SNPs were enriched for genes that represented a smaller mutational target (File S23). Consistent with this, we found a transcriptome-wide linear correlation between the number of SNPs and the length of a transcript (*r*^2^ = 0.57184; Figure 5C). While the mean length of SNP-containing transcripts was 1295, the mean length of SNP-lacking transcripts was only 480. Hence, in general, transcript length and/or the number of G (or C) nucleotides in a transcript impact the likelihood of an EMS-induced mutation. Further mutagenesis to saturate the sorghum genome may benefit from both a greater population size and a diversity of mutagens.

### Sequence context of EMS-induced SNPs

**Figure 6:**
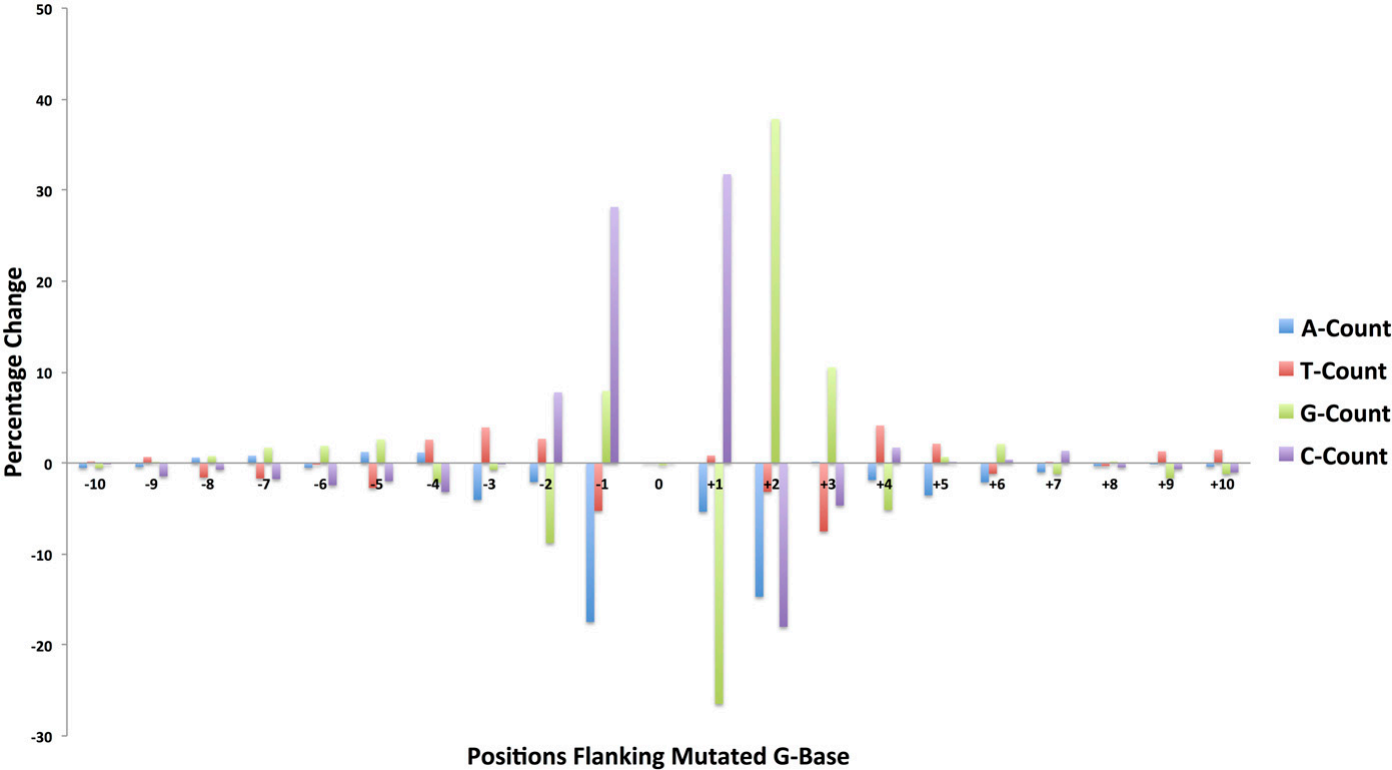
The 21 nt sequence context comparison for the mutated G-residue DNA sites in the EMS-induced SNPs and randomly selected positions in the sorghum reference genome. The percentage change in the nucleotide frequency for all DNA base types in all DNA positions shows an overrepresentation of a 5′-CGCG-3′ motif. The mutated G-residue is shown in bold. EMS, ethyl methanesulfonate; SNP, single nucleotide polymorphism.

The MEME ungapped motif search program found no single fixed length uniform motif associated with the 10,000 randomly selected SNPs. However, we observed enrichment for motifs that were present in clusters of SNPs. The most significant motif (p-value of 5.7e−304) was a 16 nt long GC-rich sequence, which is present in a cluster of only 834 SNPs out of the 10,000 homozygous SNPs (Figure S7). In the absence of uniform flanking motifs, we observed significant sequence changes in the single base immediately upstream (position −1), and two bases immediately downstream of the 608,485 mutated G-nucleotide positions, when compared to the context of random G-nucleotide positions (Figure 6). In the first base position immediately upstream of the mutated central G position, we observed a 28% increase (99,332 *vs.* 127,287) in the count of Cs, and a 17% decrease (177,307 *vs.* 146,321) in the count of A bases. At the first downstream position (+1) we observed a 32% increase (141,485 *vs.* 186,396) in the count of Cs and a 26% decrease (144,587 *vs.* 106,292) in the count of Gs. At the second downstream position (+2) we observed a 38% increase (139,477 *vs.* 192,222) in the G count and an 18% decrease (133,840 *vs.* 109,737) in the count of Cs. The reverse complement of the results of the analysis for the 606,560 mutated C reference bases yields the same pattern (Figure S8). Based on the nucleotide counts and the percentage changes at each flanking position, we observe an overrepresentation of a 5′-C**G**CG-3′ motif (with the mutagenized G position showed in bold) and an underrepresentation of 5′-AHGM-3′ [where the ambiguous DNA codes H=(A,C,T) and M=(A,C), respectively]. In comparison to the genome, the CG dinucleotide content was 57% higher in the 21 nt sequence context of the mutated positions (3.4% *vs.* 5.3%) and the 5′-C**G**CG-3′ motif is also clearly delineated in the positions 6–9 in the MEME motif results.

### Sequence context of the error-prone SNP positions

**Figure 7:**
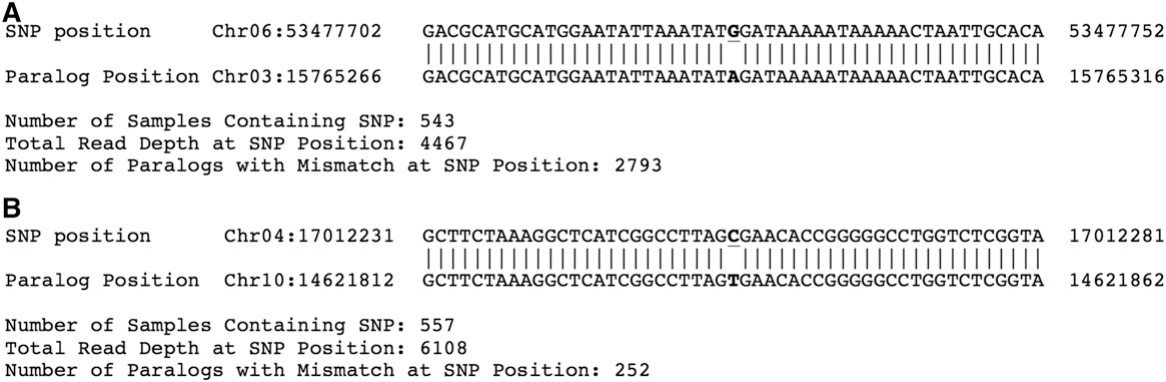
Examples of BLAST global alignments of the 51 nt sequence context of the probable error-prone SNPs to the sorghum reference genome. The sample alignments depict paralogous alignments that result in false positive G:C to A:T EMS-induced mutations. The reference and SNP positions are highlighted in bold font, with the mutated position underlined. BLAST, Basic Local Alignment Search Tool; EMS, ethyl methanesulfonate; SNP, single nucleotide polymorphism. (A) Alignment showing a G to A substitution. (B) Alignment showing a C to T substitution.

We obtained BLAST global alignment results for the 51 nt sequence context of 57,404, 55,085, and 57,994 of the error-prone SNP positions, random genomic locations, and nonreplicate EMS-induced SNP positions, respectively. Of these, 38% (21,993 positions), 37% (20,156 positions), and 24% (14,099 positions) of the error-prone, random genomic, and random EMS-induced SNPs, respectively, had perfect matching paralogous sequences in the reference genome. Considering only end-to-end ungapped alignments of these sequences, we detected a total of 68,568,482 near-perfect matches for 41,733 (73%) of the error-prone SNP positions. For the randomly selected genomic positions, we detected ungapped near-perfect matches for nearly as many loci (33,980 corresponding to 62%) but in only 29,849,107 global alignments, indicating lower copy numbers for the paralogous sequences. This pattern of lower paralog coverage for the 51 nt SNP context was also true for the nonreplicate SNPs, where we recovered threefold fewer paralogous locations on average (21,870,737 global alignments for 33,605 sites corresponding to 58%). Interestingly, 51% (34,966,393 alignments) of the ungapped alignments to error-prone SNP sequence contexts contained a mismatch at the central SNP position. In contrast, only 7% (1,996,337 alignments) and 11% (2,369,537 alignments) of the ungapped alignments for the random genomic positions and the nonreplicate EMS-induced SNPs, respectively, contained a mismatch at the central (SNP) position (Table 4). The total length of the super scaffolds that have not been assigned to any chromosome in sorghum genome reference assembly version 2.1 is 68 Mb, and accounts for 9.4% of the genome assembly. Yet, >51% (17,735,536 alignments) of the global alignments with a central mismatch in the error-prone SNP alignment data had super scaffold origins. For the nonreplicate EMS-induced SNPs and random genomic position data sets, only 32% (763,384 alignments) and 28% (563,042 alignments), respectively, of the global alignments with a central mismatch occurred in super scaffolds. The substitutions at the central mismatch positions in the global alignments matched the SNP call for 58% of the error-prone SNP positions (33,083 SNPs) but only 34.5% of the nonreplicate EMS-induced SNP positions (20,011 SNPs). The end-to-end ungapped (global) alignments may contain more than one mismatched position. Further filtering of the global alignment results to retain only alignments containing a single mismatch at the central (SNP) position identified 2,032,958 alignments for the error-prone SNP sequence contexts. Of these, 47% (951,217 alignments) were to super scaffold assemblies (File S24). Similarly, we also obtained 113,515 and 120,547 single (central) mismatched global alignments for the random genome and EMS-induced SNP data sets, respectively, but only 19 (22,011 alignments) and 28% (33,904 alignments) matched super scaffolds assemblies, respectively. This method was more stringent, and only recapitulated 26 (14,913 SNP positions) and 7% (4191 SNP positions) of the error-prone and EMS-induced SNP positions, respectively (Figure 7). These results demonstrate that paralogous sequences from poorly assembled regions of the genome match the error-prone sites detected as replicate SNPs in whole-genome sequence data from the reference genotype. The DNA represented by these assemblies disproportionally contributed to the detection of false positives in shotgun sequencing experiments.

**Table 4:** BLAST results for the global alignment of the 51 nt sequence context of the error-prone SNPs, random genomic, and random EMS-induced SNP positions

	Positions with Initial Alignments	Positions with Perfect Alignments	Number of Global Alignments	Central Mismatch in Global Alignments
Error-prone SNPs	57,404	21,993	68,568,482	34,966,393
Random genome SNPs	55,085	20,156	29,849,107	1,996,337
Random EMS-induced SNPs	57,994	14,099	21,870,737	2,369,537

SNP, single nucleotide polymorphism; EMS, ethyl methanesulfonate.

### EMS-induced variant confirmation

A total of 29 randomly selected individuals from the collection of 586 sequenced individuals were resequenced using leaf tissues from the M_5_ generation and the Illumina WideSeq paired-end sequencing protocol. The sequencing yielded mean genome coverage of 0.06×. These reads were aligned to the sorghum genome and the positions of previously identified SNPs in these 29 lines were assessed. Given that a mean of 2177 homozygous nonreplicate SNPs was found in each line, this should overlap with 130 SNPs per line and provide ample depth to test line identity. The low-depth sequence overlapped with 3843 nonreplicate SNP positions and 96% of these were unambiguously confirmed (Figure S9). This validated our SNP calling procedures, and also verified the identity of our lines and the validity of our pedigrees. There were 1745 reads that contained positions called as heterozygous nonreplicate SNPs in the M_3_ sequence data. For these heterozygous SNPs, our low-pass sequence confirmed 41% (710 out of 1745) of the mutations (Figure S10). This rate of validation again confirmed our pedigrees and line identities. The lower rate of confirmation is consistent with the expectations of segregation, where 37.5% of the M_5_ should now be homozygous references for the heterozygous position and of the 25% still heterozygous, we would recover the nonreference allele in half of the reads. This should have resulted in 50% nonreference alleles, or 48% if the validation rate was the same for heterozygous and homozygous SNPs. Consistent with the likely higher false positive rate of the heterozygous SNPs, suggested by the lower G:C to A:T SNP percentage in the nonreplicate SNPs, we validated fewer SNPs.

## Discussion

The accuracy of the *in silico* detection of sequence variants can be impacted by the inherent sequencing error rates of NGS technologies, nature of the variant-calling programs, genome complexities, sequence alignment programs, and the quality of reference genome assemblies (Estivill *et al.* 2002; Cheung *et al.* 2003; Fredman *et al.* 2004; Nielsen *et al.* 2011; O’Rawe *et al.* 2013; Cheng *et al.* 2014; Robasky *et al.* 2014). We utilized an additional step to improve current standard practices for filtering false positive variant calls detected in genetic studies using mutagenizing agents such as EMS, which induce random mutations. EMS mutagenesis should result in DNA mutations at nearly random subsets of the mutable positions in the genome of a treated organism. For an organism with a sufficiently large genome, the likelihood of a randomly induced mutation occurring at the same genomic position in two individuals that were treated independently is extremely low. Hence, a nonreplicated high-quality SNP detected at a genomic position that is unique to a single individual in a population of independently EMS-mutagenized individuals is most likely a true positive and represents a real signal in the SNP detection. If a sequencing error, on the other hand, is nonrandomly distributed, a SNP detected in multiple lines at the same genomic position is mostly likely a replicated false positive SNP. Applying this cost-effective and highly accurate filtering step to the large-scale NGS data set produced from the M_3_ generation of an EMS-mutagenized population of the agronomically important crop species *S. bicolor*, we showed that 76% of the homozygous SNPs and 94% of the heterozygous SNPs detected using standard SNP detection and filtering were false positives (Figure 1 and Figure S1). The NGS sequencing coverage depth for the 586 sorghum lines in this study was 7×, and this is lower than the 20× coverage depth often described as the minimum for accurate SNP calling (Flibotte *et al.* 2010; Liu *et al.* 2012; James *et al.* 2013). Two previous efforts had independently used a similar method to refine their variant calling (Sarin *et al.* 2010; Uchida *et al.* 2011). However, our characterization and results clearly reveals empirical evidence of the accuracy and effectiveness of the approach. We identified and cataloged the relatively few genomic positions of the sorghum reference genome, consisting of 57,872 positions, that were most probably error-prone, and which produced 4,123,621 SNP calls of high statistical quality (File S2). Similar approaches for elucidating this experimentally identifiable set of replicate SNP positions in any species, which lack the expected EMS mutation spectrum, correspond to repeated sequences, and contain paralogs with the predicted SNP, should improve the accuracy of SNP calling (Henry *et al.* 2014; Addo-Quaye *et al.* 2017). Similarly, filtering of the indel alleles retained <1% (17,760) as nonreplicate indels (Figure S4 and Tables S7 and S8 in File S26). Consistent with the analysis of indels induced by fast neutron irradiation of rice (Li G. *et al.* 2016), the vast majority of our indel alleles were short in length (2.6 bases) with sizes ranging from 1 to 50 bases. In agreement with the high false positive detection rate for indels, the recent genome-wide analysis of chemically induced-mutations obtained by N-methyl-N’-nitro-N-nitrosoguanidine mutagenesis in the social soil amoeba *Dictyostelium discoideum* also uncovered very few likely true positive alleles, while discarding 99% of initially predicted variants (Li C.-L. F. *et al.* 2016).

The mutagenic effect of EMS is well-characterized in a wide variety of viruses, prokaryotes, fungal, plant, and animal genomes (Loveless 1958, 1969; Singer *et al.* 1982; Sega 1984; Shrivastav *et al.* 2010). The predominant DNA reactive site that produces DNA lesions after EMS alkylation is O^6^ guanine. The ethylation of O^6^ guanine results in low mutagenicity in the EMS-treated organism (Loveless 1969; Singer *et al.* 1982). We found an average of only 2177 homozygous and 815 heterozygous nonreplicate SNP calls per sorghum individual (Table 2). Overall, 27% of the nonreplicate SNPs were heterozygous, and this was slightly higher than the expected ∼22% heterozygosity for an M_3_ population. Two possible dynamics contributed to the minor discrepancy between the observed and expected levels of SNP heterozygosity: false positives due to *in silico* overestimation and *bona fide* heterozygous calls due to pollen contamination. In contrast to the homozygous SNP predictions, we observed a positive correlation (*R* = 0.51) between the number of *in silico* heterozygous SNPs detected and the read mapping coverage for the sequenced individuals. In attempting to compensate for a lack of sufficient sequencing coverage for predicting the heterozygous SNPs, our relaxed computational stringency for predicting heterozygous SNPs may have caused an overestimation in the number of heterozygous calls. This explanation applies more specifically to samples with lower sequencing coverage. For some individuals, the consequence of pollen contamination from outcrossing to previous generations, and not sequencing coverage, may have caused an increase in the level of heterozygosity. For example, 21 of the sequenced individuals with at least 7× sequencing coverage were at most only 20% homozygous (Figure 3). The mispairing of O^6^ alkyl guanine with thymine results in G:C to A:T mutations (Loveless 1969; Prakash and Sherman 1973; Kohalmi and Kunz 1988). The mutation spectra of the detected homozygous and heterozygous nonreplicate SNPs showed that 98 and 94% were G:C to A:T transitions, respectively (Figure S2). The slightly lower percentage of G:C to A:T transitions for the heterozygous SNPs may be attributed to higher false positive rates due to the inclusion of a greater number of sites with paralogous positions within the heterozygous SNP class, as well as the lower overall stringency when non-SNP containing reads are allowed. The notion that we still have some false positives within our SNP calls is supported by the fact that within the protein-coding regions >99% of the homozygous SNPs that we detected were G:C to A:T substitutions. These positions are less likely to possess unassembled paralogous sequences than the intergenic positions. This frequency matches a previous study in *Arabidopsis* that found that >99% of EMS-induced mutations in a set of 192 genes were G:C to A:T substitutions (Greene *et al.* 2003). Although DNA lesions from EMS mutagenesis are predominantly associated with O^6^ guanine ethylation, other minor sites such as O^2^ and O^4^ thymine had also been suggested. Studies show that the alkylation of O^4^ thymine results in mispairing with guanine and that this leads to A:T to G:C transitions (Prakash and Sherman 1973; Coulondre and Miller 1977; Singer *et al.* 1978). Our analysis revealed that A:T to G:C transitions were the second most abundant class of likely EMS-induced mutations and accounted for 0.7% of the homozygous SNPs. This observation corroborates the finding in viruses that suggests that EMS produces A:T to G:C transitions at almost two orders of magnitude less frequently than G:C to A:T transitions (Krieg 1963).

Previous large-scale analysis of flanking positions of predicted EMS-induced mutations in sorghum reported an overrepresentation of cytosine residues at positions −2 and +1 (Jiao *et al.* 2016). In addition to these sites, we detected a more extensive signal characterized by a genome-wide overrepresentation of a 5′-CGCG-3′ motif and an underrepresentation of a 5′-AHGM-3′ motif (Figure 6, Figure S7, and Figure S8). In particular, the intriguing upsurge in the C count occurring at the position immediately preceding the mutated G implied a greater proportion of CG dinucleotides among the mutated bases. These dinucleotides are underrepresented (3.6%) in the sorghum genome (version 2.1), and are the sites of DNA methylation. Methylated cytosines are known to spontaneously deaminate to thymidine. For this reason, the G to A mutational impact of EMS, which would pair perfectly with thymidine, may be more readily affected at these locations and go undetected by repair processes. We observed that the CG dinucleotide content in the 21 nt context of the mutation was 57% higher than that of the sorghum reference genome. The observation that a similar sequence occurs as an overrepresented motif was described previously in the large-scale exome sequencing of rice EMS mutants (Henry *et al.* 2014). This observation suggests that something in common between the two systems, either inherent to the structure of the DNA sequence, the repair process, or the methylation of cytosines, is responsible for the higher rate of mutation. Whereas the rice EMS-induced mutations were more likely at unmethylated cytosines (Henry *et al.* 2014), sorghum EMS-induced mutations were overrepresented at methylated cytosines (Jiao *et al.* 2016). It is important to note that, while the relationships between cytosine methylation and EMS-induced mutations were in opposite directions in rice and sorghum, methylation did not affect the majority of positions.

Since EMS mutagenesis targets predominantly random G positions in the genome, we found that the number of mutations in a gene is correlated with the gene length and the G+C count, but not with GC content. Hence, we observed that, in the absence of saturation, short gene transcripts with fewer G:C sites were likely to have few or no mutations in our population. Due to the nucleotide composition of the three canonical translational stop codons (TAA, TGA, and TAG), and the fact that the majority of the likely EMS-induced mutations are G:C to A:T substitutions, stop codons are primarily buffered from the effects of EMS mutagenesis and we saw very few losses of stop codons. TAA codons do not contain any G and/or C nucleotide targets of EMS, while the G to A substitution of TGA or TAG converts them silently to TAA stop codons. We did observe 148 such silent stop codon substitutions in our SNP annotation (Table S6 in File S26), which may be of use to others interested in the regulation of stop codons, the contribution of stop codon usage to mRNA turnover, or nonsense suppression. We observed a single homozygous and four heterozygous stop codon loss SNPs that were due to non-G:C to A:T substitutions that should result in translation read-through (Table 3).

Previous studies have revealed the potential for genome assembly errors in the presence of repetitive sequences and segmental duplications that result in false positive or ambiguous variants (Estivill *et al.* 2002; Cheung *et al.* 2003; Fredman *et al.* 2004). Our examination of the sequence context of the identified error-prone positions showed evidence that paralogous sequence variants, with origins in repetitive and duplicated genomic regions, contributed to false positive detections. We observed that the replicate SNPs were present in poorly assembled regions of the genome and present in a greater number of paralogs. The nonreplicate SNP positions were drawn from a dramatically lower proportion of positions with perfect match paralogs in the genome than expected by random chance (24% in the nonreplicate SNPs but 37% of randomly selected genomic locations). This demonstrates that SNP calling in polyploids, even paleopolyploids, will be suppressed at positions of conservation by ambiguous alignment and the erroneous assessment of no variation. Special procedures for calling SNPs at paralogs should improve SNP detection in subsets of genomes characterized by identity. Another source of the replicated SNP calls could be spontaneous mutations that arose in the parental line used in the mutagenesis, but that were absent in the reference genome genotype. For example, a recent resequencing of the sorghum BTx623 parental line utilized in a mutagenesis experiment revealed 13,243 high-confidence variations when compared to the reference sorghum genotype (Jiao *et al.* 2016). We found that the average sequence coverage depths at the error-prone SNP positions and the *bona fide* SNP position profiles were not different in our data. This may be due to the randomized assignment of nonunique reads from repeated sequences to each repeat location by the BWA aligner (Firtina and Alkan 2016). Novel methods for improving the assignment of multi-mapping reads are emerging and may improve SNP calling when widely implemented (Johnson *et al.* 2016).

Although the removal of the shared variants is a very effective technique for identifying likely false positive EMS-induced mutations, a few caveats need to be highlighted. First, the method should be applied meticulously to prevent the introduction of false negatives. For example, sample mislabeling and unexpected hybridization between individuals within the population can result in the sequencing of multiple DNA samples containing shared (identity by descent) mutagenized chromosomes. Sequence contamination originating from sample or library preparation stages can also negatively impact the detection of false positives. In all the above-mentioned scenarios, technical errors or contaminants would result in the erroneous purging of true DNA variants that were shared due to identity by descent from a common ancestor. In contrast to noncontaminated samples, prior to population-wide subtraction of shared variations, sample pairs connected by contamination are characterized by an abnormally high percentage of shared variation between the two individuals. Prior to the removal of common variants, pairwise comparison of detected variants in all the sequenced individuals in the population is mandated to expose contaminated samples with shared pedigrees, regardless of how certain biologists are that there is a lack of contamination in their work. Second, although the likelihood is extremely low, it is not statistically impossible for a mutation to be independently induced at the same genomic position in two or more individuals (Krasileva *et al.* 2017). Hence, it is noteworthy that there is a possibility that a few replicate variants are real. This frequency increases as the sample size and mutation density go up, and as mutations are nonrandomly distributed in the genome. A careful examination of the subtracted replicate SNPs could recover false negative SNPs with origins in potential mutation hotspots. Our subsequent signal-to-noise analysis of the replicate SNPs predicted at the error-prone genomic positions found a nontrivial (G:C to A:T substitution percentage) signal for the cluster of genomic positions where the same SNP was predicted in at most two individuals (see *Materials and Methods* section; Figure S3 and Table S4 in File S26). Hence, we selected an additional 18,582 homozygous and 11,418 heterozygous replicates or nonunique SNPs as likely false negative coincidental SNPs.

In biological experiments, replication plays a critical role by adding statistical power to both detect differences and reject errant stochastic results, as discussed previously for NGS experiments (Robasky *et al.* 2014). Based on our work in a homozygous model system, we reckon that anyone applying NGS sequencing to identify a causal variant in the sequencing data from a single mutant line would likely have to sort through a vast majority of false positives. In the absence of intersample variant comparisons, the majority of the standard filtered SNPs in each line will appear to be possible causative mutations. If the same line were to be resequenced, it would return a subset of the false positive calls a second time, meeting the requirement of reproducibility, and these will be falsely confirmed as true DNA variants. As such we are skeptical of rare variant detection claims that have not sought rigorous explanations for the repeated detection of SNPs in experimental samples. For instance, the relevance of the error-prone positions to the cataloging of rare variants induced during cancer progression is needed. Hence, the quality of reference genome assemblies, the complexity of genomes, and a careful, critical view of NGS data demonstrate the need for a more sophisticated approach to false positive detection when rare variant identification is the experimental goal.

### Conclusions

The prevalence of false positive DNA variants negatively impacts the efficiency of forward and reverse genetics studies. We utilized and described a cost-effective, high-throughput, and highly accurate NGS method for improving the *in silico* detection of induced mutations via the identification of false positive variants. This method is generally applicable to any genetics study and is also independent of the choice of the variant-calling program. Using the method, we have detected and described the set of EMS-induced mutations in a population of *S. bicolor*, which is a next-generation crop species with tremendous agronomical potential. We hope that the genetics community and plant breeders will find the described genetic resource to be very useful.

## Supplementary Material

Supplemental material is available online at www.g3journal.org/lookup/suppl/doi:10.1534/g3.117.300301/-/DC1.

## References

[bib1] Addo-Quaye C. Buescher E. Best N. Chaikam V. Baxter I., 2017 Forward genetics by sequencing EMS variation-induced inbred lines. G3 (Bethesda) 7: 413–425. 28040779 10.1534/g3.116.029660 PMC5295590

[bib2] Alonso J. M. Ecker J. R., 2006 Moving forward in reverse: genetic technologies to enable genome-wide phenomic screens in *Arabidopsis*. Nat. Rev. Genet. 7: 524–536. 16755288 10.1038/nrg1893

[bib3] Altschul S. F. Gish W. Miller W. Myers E. W. Lipman D. J., 1990 Basic local alignment search tool. J. Mol. Biol. 215: 403–410. 2231712 10.1016/S0022-2836(05)80360-2

[bib4] Arumuganathan K. Earle E. D., 1991 Nuclear DNA content of some important plant species. Plant Mol. Biol. Report. 9: 208–218.

[bib5] Bailey T. L. Boden M. Buske F. Frith M. Grant C. E., 2009 MEME SUITE: tools for motif discovery and searching. Nucleic Acids Res. 37: W202–W208. 19458158 10.1093/nar/gkp335 PMC2703892

[bib6] Bevan M. W. Uauy C. Wulff B. B. H. Zhou J. Krasileva K., 2017 Genomic innovation for crop improvement. Nature 543: 346–354. 28300107 10.1038/nature22011

[bib7] Blomstedt C. K. Gleadow R. M. O’Donnell N. Naur P. Jensen K., 2012 A combined biochemical screen and TILLING approach identifies mutations in Sorghum bicolor L. Moench resulting in acyanogenic forage production. Plant Biotechnol. J. 10: 54–66. 21880107 10.1111/j.1467-7652.2011.00646.x

[bib8] Camacho C. Coulouris G. Avagyan V. Ma N. Papadopoulos J., 2009 BLAST+: architecture and applications. BMC Bioinformatics 10: 421. 20003500 10.1186/1471-2105-10-421 PMC2803857

[bib9] Cheng A. Y. Teo Y.-Y. Ong R. T.-H., 2014 Assessing single nucleotide variant detection and genotype calling on whole-genome sequenced individuals. Bioinformatics 30: 1707–1713. 24558117 10.1093/bioinformatics/btu067

[bib10] Cheung J. Estivill X. Khaja R. MacDonald J. R. Lau K., 2003 Genome-wide detection of segmental duplications and potential assembly errors in the human genome sequence. Genome Biol. 4: R25. 12702206 10.1186/gb-2003-4-4-r25 PMC154576

[bib11] Cingolani P. Patel V. M. Coon M. Nguyen T. Land S. J., 2012 a Using *Drosophila melanogaster* as a model for genotoxic chemical mutational studies with a new program, SnpSift. Front. Genet. 3: 35. 22435069 10.3389/fgene.2012.00035 PMC3304048

[bib12] Cingolani P. Platts A. Wang L. L. Coon M. Nguyen T., 2012 b A program for annotating and predicting the effects of single nucleotide polymorphisms, SnpEff: SNPs in the genome of Drosophila melanogaster strain w1118; iso-2; iso-3. Fly (Austin) 6: 80–92. 22728672 10.4161/fly.19695 PMC3679285

[bib13] Coulondre C. Miller J. H., 1977 Genetic studies of the lac repressor. IV. Mutagenic specificity in the lacI gene of *Escherichia coli*. J. Mol. Biol. 117: 577–606. 416218 10.1016/0022-2836(77)90059-6

[bib14] Danecek P. Auton A. Abecasis G. Albers C. Banks E., 2011 The variant call format and VCFtools. Bioinformatics 27: 2156–2158. 21653522 10.1093/bioinformatics/btr330 PMC3137218

[bib15] DePristo M. Banks E. Poplin R. Garimella K. V. Maguire J. R., 2011 A framework for variation discovery and genotyping using next-generation DNA sequencing data. Nat. Genet. 43: 491–498. 21478889 10.1038/ng.806 PMC3083463

[bib16] Estivill X. Cheung J. Pujana M. A. Nakabayashi K. Scherer S. W., 2002 Chromosomal regions containing high-density and ambiguously mapped putative single nucleotide polymorphisms (SNPs) correlate with segmental duplications in the human genome. Hum. Mol. Genet. 11: 1987–1995. 12165560 10.1093/hmg/11.17.1987

[bib17] Firtina C. Alkan C., 2016 On genomic repeats and reproducibility. Bioinformatics 32: 2243–2247. 27153582 10.1093/bioinformatics/btw139

[bib18] Flibotte S. Edgley M. L. Chaudhry I. Taylor J. Neil S. E., 2010 Whole-genome profiling of mutagenesis in *Caenorhabditis elegans*. Genetics 185: 431–441. 20439774 10.1534/genetics.110.116616 PMC2881127

[bib19] Fredman D. White S. J. Potter S. Eichler E. E. Den Dunnen J. T., 2004 Complex SNP-related sequence variation in segmental genome duplications. Nat. Genet. 36: 861–866. 15247918 10.1038/ng1401

[bib20] Goodstein D. M. Shu S. Howson R. Neupane R. Hayes R. D., 2012 Phytozome: a comparative platform for green plant genomics. Nucleic Acids Res. 40: D1178–D1186. 22110026 10.1093/nar/gkr944 PMC3245001

[bib21] Greene E. A. Codomo C. A. Taylor N. E. Henikoff J. G. Till B. J., 2003 Spectrum of chemically induced mutations from a large-scale reverse-genetic screen in Arabidopsis. Genetics 164: 731–740. 12807792 10.1093/genetics/164.2.731 PMC1462604

[bib22] Grierson C. S. Barnes S. R. Chase M. W. Clarke M. Grierson D., 2011 One hundred important questions facing plant science research. New Phytol. 192: 6–12. 21883238 10.1111/j.1469-8137.2011.03859.x

[bib23] Henry I. M. Nagalakshmi U. Lieberman M. C. Ngo K. J. Krasileva K. V., 2014 Efficient genome-wide detection and cataloging of EMS-induced mutations using exome capture and next-generation sequencing. Plant Cell 26: 1382–1397. 24728647 10.1105/tpc.113.121590 PMC4036560

[bib24] James G. V. Patel V. Nordström K. J. Klasen J. R. Salomé P. A., 2013 User guide for mapping-by-sequencing in *Arabidopsis*. Genome Biol. 14: R61. 23773572 10.1186/gb-2013-14-6-r61 PMC3706810

[bib25] Jiao Y. Burke J. Chopra R. Burow G. Chen J., 2016 A Sorghum mutant resource as an efficient platform for gene discovery in grasses. Plant Cell 28: 1551–1562. 27354556 10.1105/tpc.16.00373 PMC4981137

[bib26] Johnson N. R. Yeoh J. M. Coruh C. Axtell M. J., 2016 Improved placement of multi-mapping small RNAs. G3 (Bethesda) 6: 2103–2111. 27175019 10.1534/g3.116.030452 PMC4938663

[bib27] Koegel S. Ait Lahmidi N. Arnould C. Chatagnier O. Walder F., 2013 The family of ammonium transporters (AMT) in *Sorghum bicolor*: two AMT members are induced locally, but not systemically in roots colonized by arbuscular mycorrhizal fungi. New Phytol. 198: 853–865. 23461653 10.1111/nph.12199

[bib28] Kohalmi S. E. Kunz B., 1988 Role of neighbouring bases and assessment of strand specificity in ethylmethanesulphonate and *N*-methyl-*N*′-nitro-*N*-nitrosoguanidine mutagenesis in the *SUP*4-o gene of *Saccharomyces cerevisiae*. J. Mol. Biol. 204: 561–568. 3066906 10.1016/0022-2836(88)90355-5

[bib29] Krasileva K. V. Vasquez-Gross H. A. Howell T. Bailey P. Paraiso F., 2017 Uncovering hidden variation in polyploid wheat. Proc. Natl. Acad. Sci. USA 114: E913–E921. 28096351 10.1073/pnas.1619268114 PMC5307431

[bib30] Krieg D. R., 1963 Ethyl methanesulfonate-induced reversion of bacteriophage T4rII mutants. Genetics 48: 561–580. 14035786 10.1093/genetics/48.4.561 PMC1210494

[bib31] Krothapalli K. Buescher E. M. Li X. Brown E. Chapple C., 2013 Forward genetics by genome sequencing reveals that rapid cyanide release deters insect herbivory of *Sorghum bicolor*. Genetics 195: 309–318. 23893483 10.1534/genetics.113.149567 PMC3781961

[bib32] Kumar P. Henikoff S. Ng P. C., 2009 Predicting the effects of coding non-synonymous variants on protein function using the SIFT algorithm. Nat. Protoc. 4: 1073–1081. 19561590 10.1038/nprot.2009.86

[bib33] Li C.-L. F. Santhanam B. Webb A. N. Zupan B. Shaulsky G., 2016 Gene discovery by chemical mutagenesis and whole-genome sequencing in *Dictyostelium*. Genome Res. 26: 1268–1276. 27307293 10.1101/gr.205682.116 PMC5052037

[bib34] Li G. Chern M. Jain R. Martin J. A. Schackwitz W. S., 2016 Genome-wide sequencing of 41 rice (*Oryza sativa* L.) mutated lines reveals diverse mutations induced by fast-neutron irradiation. Mol. Plant 9: 1078–1081. 27018389 10.1016/j.molp.2016.03.009

[bib35] Li H. Durbin R., 2009 Fast and accurate short read alignment with Burrows-Wheeler transform. Bioinformatics 25: 1754–1760. 19451168 10.1093/bioinformatics/btp324 PMC2705234

[bib36] Li H. Handsaker B. Wysoker A. Fennell T. Ruan J., 2009 The sequence alignment/map format and SAMtools. Bioinformatics 25: 2078–2079. 19505943 10.1093/bioinformatics/btp352 PMC2723002

[bib37] Liu K. McCormack M. Sheen J., 2012 Targeted parallel sequencing of large genetically-defined genomic regions for identifying mutations in *Arabidopsis*. Plant Methods 8: 12. 22462410 10.1186/1746-4811-8-12 PMC3348062

[bib38] Loveless A., 1958 Increased rate of plaque-type and host-range mutation following treatment of bacteriophage *in vitro* with ethyl methane sulphonate. Nature 181: 1212–1213. 13541432 10.1038/1811212a0

[bib39] Loveless A., 1969 Possible relevance of O-6 alkylation of deoxyguanosine to the mutagenicity and carcinogenicity of nitrosamines and nitrosamides. Nature 223: 206–207. 5791738 10.1038/223206a0

[bib40] Metzker M. L., 2010 Sequencing technologies - the next generation. Nat. Rev. Genet. 11: 31–46. 19997069 10.1038/nrg2626

[bib41] Nakamura K. Oshima T. Morimoto T. Ikeda S. Yoshikawa H., 2011 Sequence-specific error profile of Illumina sequencers. Nucleic Acids Res. 39: e90. 21576222 10.1093/nar/gkr344 PMC3141275

[bib42] Nielsen R. Paul J. S. Albrechtsen A. Song Y. S., 2011 Genotype and SNP calling from next-generation sequencing data. Nat. Rev. Genet. 12: 443–451. 21587300 10.1038/nrg2986 PMC3593722

[bib43] Nordborg M. Weigel D., 2008 Next-generation genetics in plants. Nature 456: 720–723. 19079047 10.1038/nature07629

[bib44] Nordström K. J. V. Albani M. C. James G. V. Gutjahr C. Hartwig B., 2013 Mutation identification by direct comparison of whole-genome sequencing data from mutant and wild-type individuals using k-mers. Nat. Biotechnol. 31: 325–330. 23475072 10.1038/nbt.2515

[bib45] O’Rawe J. Jiang T. Sun G. Wu Y. Wang W., 2013 Low concordance of multiple variant-calling pipelines: practical implications for exome and genome sequencing. Genome Med. 5: 28. 23537139 10.1186/gm432 PMC3706896

[bib46] Page D. R. Grossniklaus U., 2002 The art and design of genetic screens: Arabidopsis thaliana. Nat. Rev. Genet. 3: 124–136. 11836506 10.1038/nrg730

[bib47] Paterson A. H., 2008 Genomics of sorghum. Int. J. Plant Genomics 2008: 362451. 18483564 10.1155/2008/362451 PMC2375965

[bib48] Paterson A. H. Bowers J. E. Bruggmann R. Dubchak I. Grimwood J., 2009 The *Sorghum bicolor* genome and the diversification of grasses. Nature 457: 551–556. 19189423 10.1038/nature07723

[bib49] Pedersen J. F. Bean S. R. Graybosch R. Park S. H. Tilley M., 2005 Characterization of waxy grain sorghum lines in relation to granule-bound starch synthase. Euphytica 144: 151–156.

[bib50] Peters P. J. Jenks M. Rich P. J. Axtell J. D. Ejeta G., 2009 Mutagenesis, selection, and allelic analysis of epicuticular wax mutants in Sorghum. Crop Sci. 49: 1250.

[bib51] Peterson D. G. Schulze S. R. Sciara E. B. Lee S. A. Bowers J. E., 2002 Integration of Cot analysis, DNA cloning, and high-throughput sequencing facilitates genome characterization and gene discovery. Genome Res. 12: 795–807. 11997346 10.1101/gr.226102 PMC186575

[bib52] Petti C. Harman-Ware A. E. Tateno M. Kushwaha R. Shearer A., 2013 Sorghum mutant RG displays antithetic leaf shoot lignin accumulation resulting in improved stem saccharification properties. Biotechnol. Biofuels 6: 146. 24103129 10.1186/1754-6834-6-146 PMC3852544

[bib53] Petti C. Hirano K. Stork J. DeBolt S., 2015 Mapping of a cellulose-deficient mutant named dwarf1–1 in Sorghum bicolor to the green revolution gene gibberellin20-oxidase reveals a positive regulatory association between gibberellin and cellulose biosynthesis. Plant Physiol. 169: 705–716. 26198258 10.1104/pp.15.00928 PMC4577427

[bib54] Prakash L. Sherman F., 1973 Mutagenic specificity: reversion of iso-1-cytochrome c mutants of yeast. J. Mol. Biol. 79: 65–82. 4355598 10.1016/0022-2836(73)90270-2

[bib55] Price H. J. Dillon S. L. Hodnett G. Rooney W. L. Ross L., 2005 Genome evolution in the genus Sorghum (Poaceae). Ann. Bot. 95: 219–227. 15596469 10.1093/aob/mci015 PMC4246720

[bib56] Quinlan A. R. Hall I. M., 2010 BEDTools: a flexible suite of utilities for comparing genomic features. Bioinformatics 26: 841–842. 20110278 10.1093/bioinformatics/btq033 PMC2832824

[bib57] Rizal G. Thakur V. Dionora J. Karki S. Wanchana S., 2015 Two forward genetic screens for vein density mutants in sorghum converge on a cytochrome P450 gene in the brassinosteroid pathway. Plant J. 84: 257–266. 26333774 10.1111/tpj.13007

[bib58] Robasky K. Lewis N. E. Church G. M., 2014 The role of replicates for error mitigation in next-generation sequencing. Nat. Rev. Genet. 15: 56–62. 24322726 10.1038/nrg3655 PMC4103745

[bib59] Rowan B. A. Weigel D. Koenig D., 2011 Developmental genetics and new sequencing technologies: the rise of nonmodel organisms. Dev. Cell 21: 65–76. 21763609 10.1016/j.devcel.2011.05.021

[bib60] Sarin S. Bertrand V. Bigelow H. Boyanov A. Doitsidou M., 2010 Analysis of multiple ethyl methanesulfonate-mutagenized *Caenorhabditis elegans* strains by whole-genome sequencing. Genetics 185: 417–430. 20439776 10.1534/genetics.110.116319 PMC2881126

[bib61] Sattler S. E. Saballos A. Xin Z. Funnell-Harris D. L. Vermerris W., 2014 Characterization of novel Sorghum *brown midrib* mutants from an EMS-mutagenized population. G3 (Bethesda) 4: 2115–2124. 25187038 10.1534/g3.114.014001 PMC4232537

[bib62] Schlötterer C. Tobler R. Kofler R. Nolte V., 2014 Sequencing pools of individuals—mining genome-wide polymorphism data without big funding. Nat. Publ. Gr. 15: 749–763. 10.1038/nrg3803 25246196

[bib63] Schneeberger K., 2014 Using next-generation sequencing to isolate mutant genes from forward genetic screens. Nat. Rev. Genet. 15: 662–676. 25139187 10.1038/nrg3745

[bib64] Schneeberger K. Weigel D., 2011 Fast-forward genetics enabled by new sequencing technologies. Trends Plant Sci. 16: 282–288. 21439889 10.1016/j.tplants.2011.02.006

[bib65] Scully E. D. Gries T. Funnell-Harris D. L. Xin Z. Kovacs F. A., 2015 Characterization of novel *Brown midrib 6* mutations affecting lignin biosynthesis in sorghum. J. Integr. Plant Biol. 58: 136–149. 26172142 10.1111/jipb.12375

[bib66] Sega G. A., 1984 A review of the genetic effects of ethyl methanesulfonate. Mutat. Res. 134: 113–142. 6390190 10.1016/0165-1110(84)90007-1

[bib67] Shrivastav N. Li D. Essigmann J. M., 2010 Chemical biology of mutagenesis and DNA repair: cellular responses to DNA alkylation. Carcinogenesis 31: 59–70. 19875697 10.1093/carcin/bgp262 PMC2802671

[bib68] Singer B. Fraenkel-Conrat H. Kuśmierek J. T., 1978 Preparation and template activities of polynucleotides containing O2- and O4-alkyluridine. Proc. Natl. Acad. Sci. USA 75: 1722–1726. 273902 10.1073/pnas.75.4.1722 PMC392411

[bib69] Singer B. Kuśmierek J. T. Ku J. T., 1982 Chemical mutagenesis. Annu. Rev. Biochem. 51: 655–691. 7051963 10.1146/annurev.bi.51.070182.003255

[bib70] Suzek B. E. Huang H. McGarvey P. Mazumder R. Wu C. H., 2007 UniRef: comprehensive and non-redundant UniProt reference clusters. Bioinformatics 23: 1282–1288. 17379688 10.1093/bioinformatics/btm098

[bib71] Swigoňová Z. Lai J. Ma J. Ramakrishna W. Llaca V., 2004 Close split of sorghum and maize genome progenitors. Genome Res. 14: 1916–1923. 15466289 10.1101/gr.2332504 PMC524415

[bib72] Uchida N. Sakamoto T. Kurata T. Tasaka M., 2011 Identification of EMS-induced causal mutations in a non-reference *Arabidopsis thaliana* accession by whole genome sequencing. Plant Cell Physiol. 52: 716–722. 21398646 10.1093/pcp/pcr029

[bib73] UniProt Consortium, 2014 Activities at the Universal Protein Resource (UniProt). Nucleic Acids Res. 42: D191–D198. 24253303 10.1093/nar/gkt1140 PMC3965022

[bib74] Waugh R. Leader D. J. McCallum N. Caldwell D., 2006 Harvesting the potential of induced biological diversity. Trends Plant Sci. 11: 71–79. 16406304 10.1016/j.tplants.2005.12.007

[bib75] Westergaard M., 1957 Chemical mutagenesis in relation to the concept of the gene. Experientia 13: 224–234. 13447934 10.1007/BF02157427

[bib76] Wu Y. Yuan L. Guo X. Holding D. R. Messing J., 2013 Mutation in the seed storage protein kafirin creates a high-value food trait in sorghum. Nat. Commun. 4: 2217. 23948869 10.1038/ncomms3217

[bib78] Xin Z. Chen J., 2012 A high throughput DNA extraction method with high yield and quality. Plant Methods 8: 26. 22839646 10.1186/1746-4811-8-26 PMC3441248

[bib77] Xin Z. Wang M. L. Burow G. Burke J., 2009 An induced Sorghum mutant population suitable for bioenergy research. BioEnergy Res. 2: 10–16.

