# Supplementary figures and images for: Whole-Genome Sequence Accuracy Is Improved by Replication in a Population of Mutagenized Sorghum
Source: G3 (Bethesda). 2018 Jan 25;8(3):1079–94. doi: 10.1534/g3.117.300301 (PMC5844295; doi:10.1534/g3.117.300301)

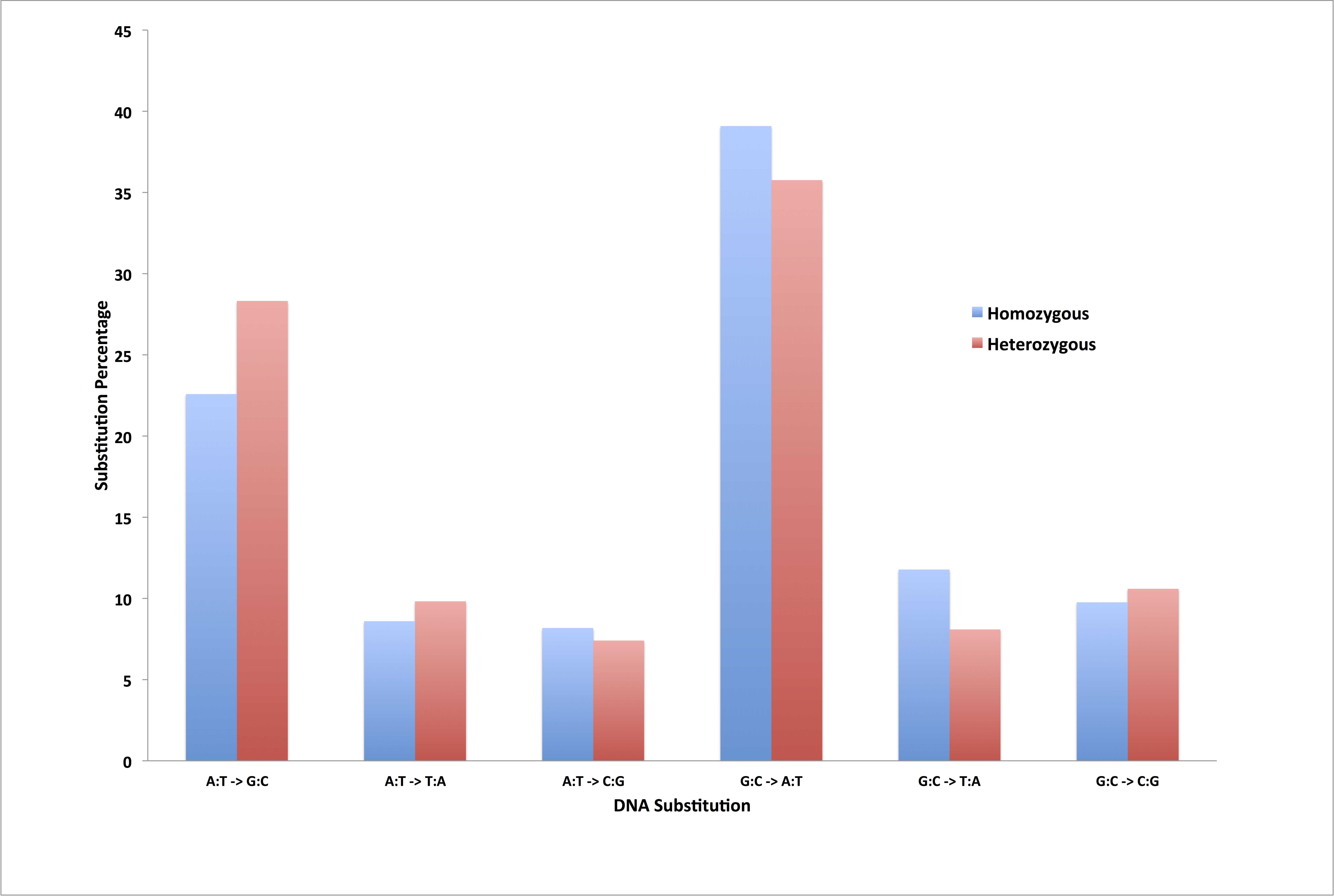

Supplement: Supplementary file 1 [file 1079FigureS1.jpg]

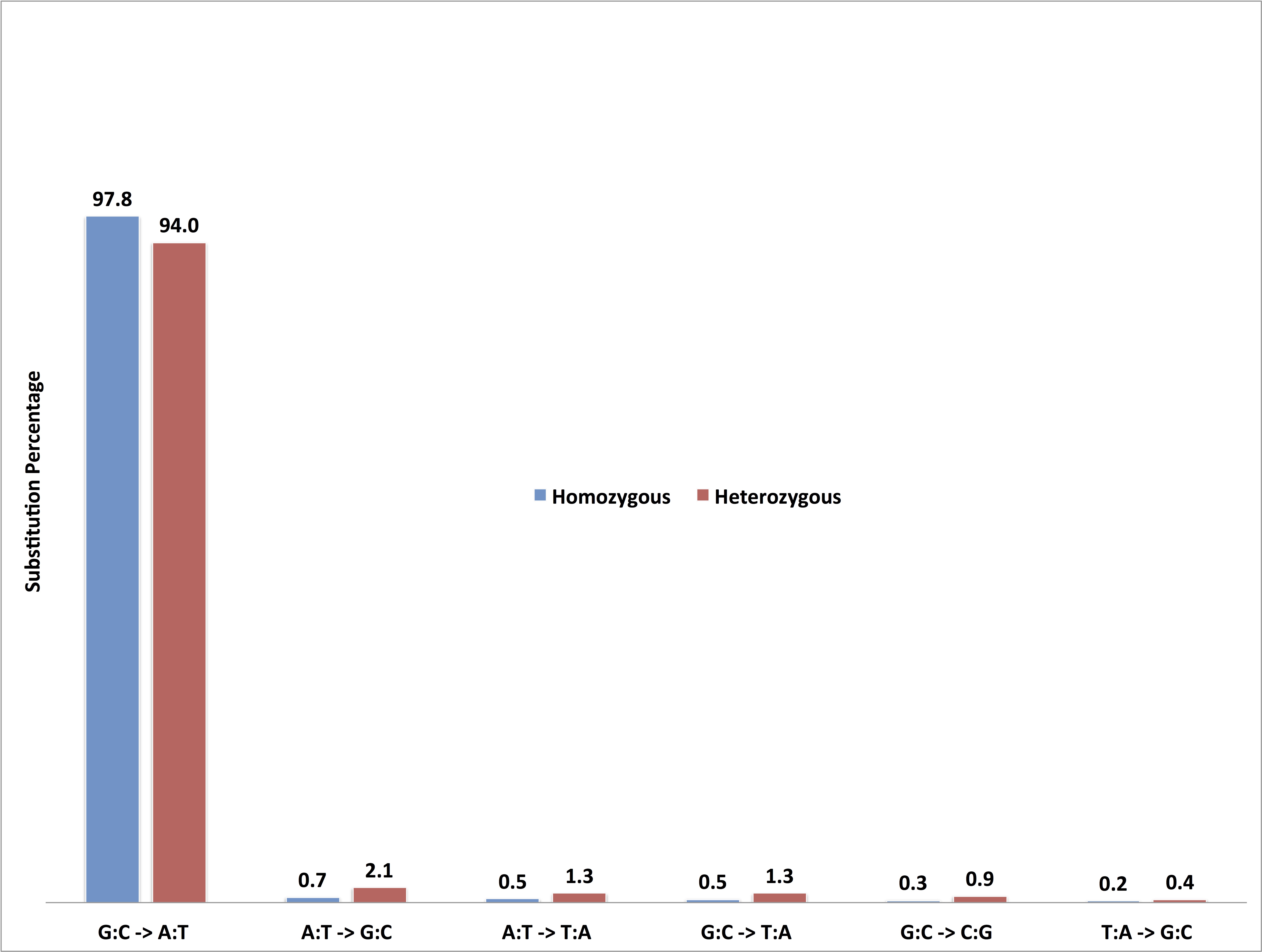

Supplement: Supplementary file 2 [file 1079FigureS2.jpg]

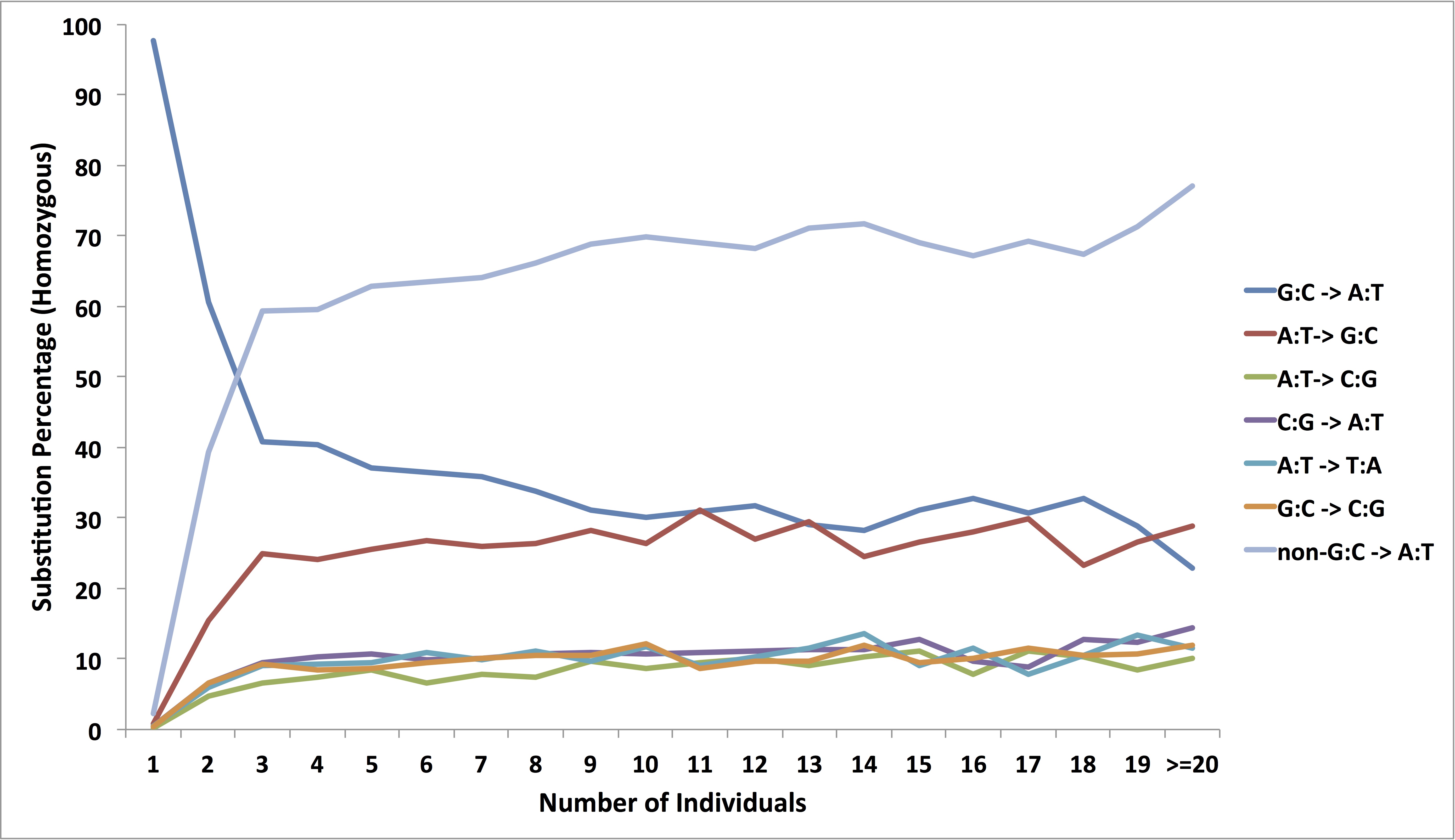

Supplement: Supplementary file 3 [file 1079FigureS3.jpg]

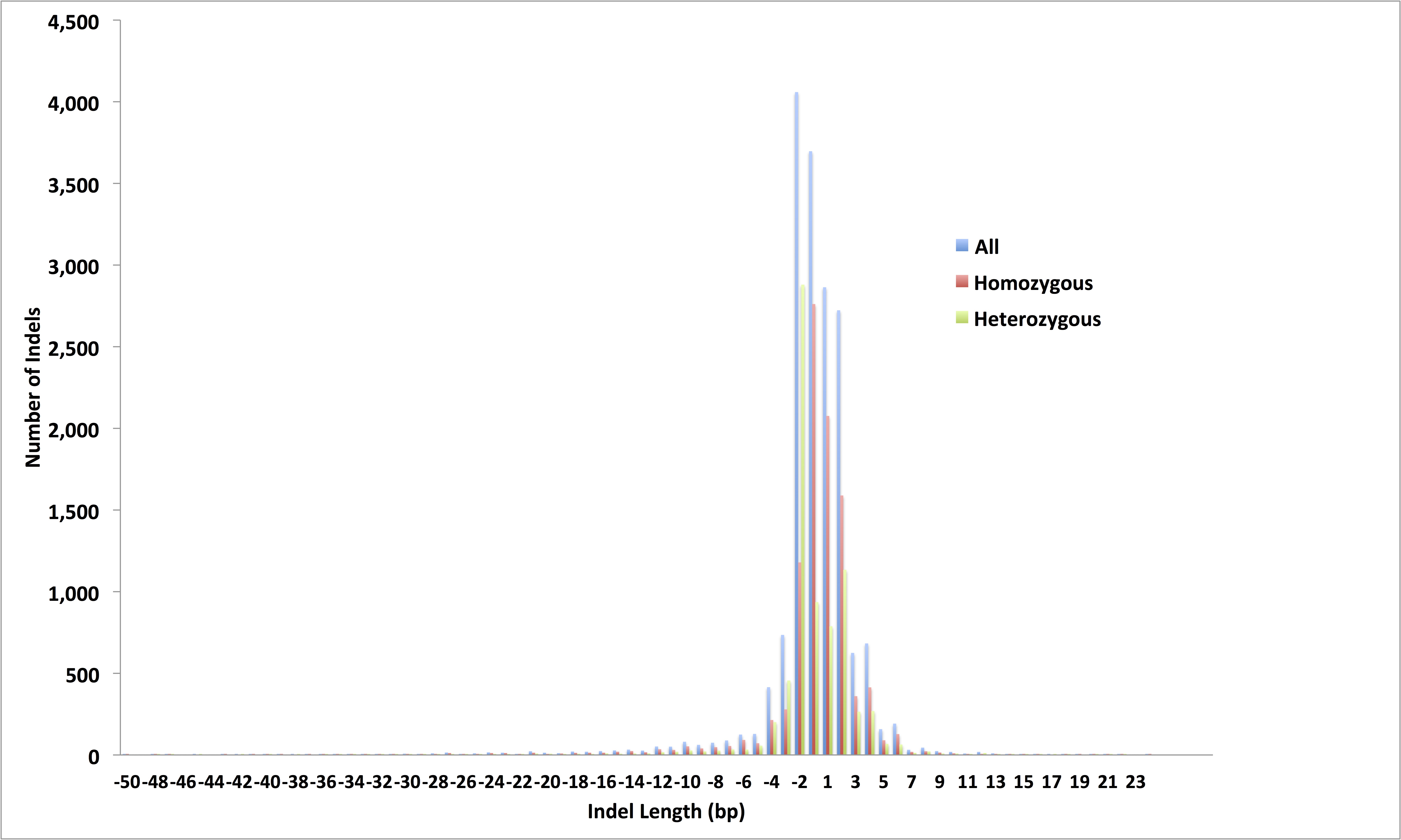

Supplement: Supplementary file 4 [file 1079FigureS4.jpg]

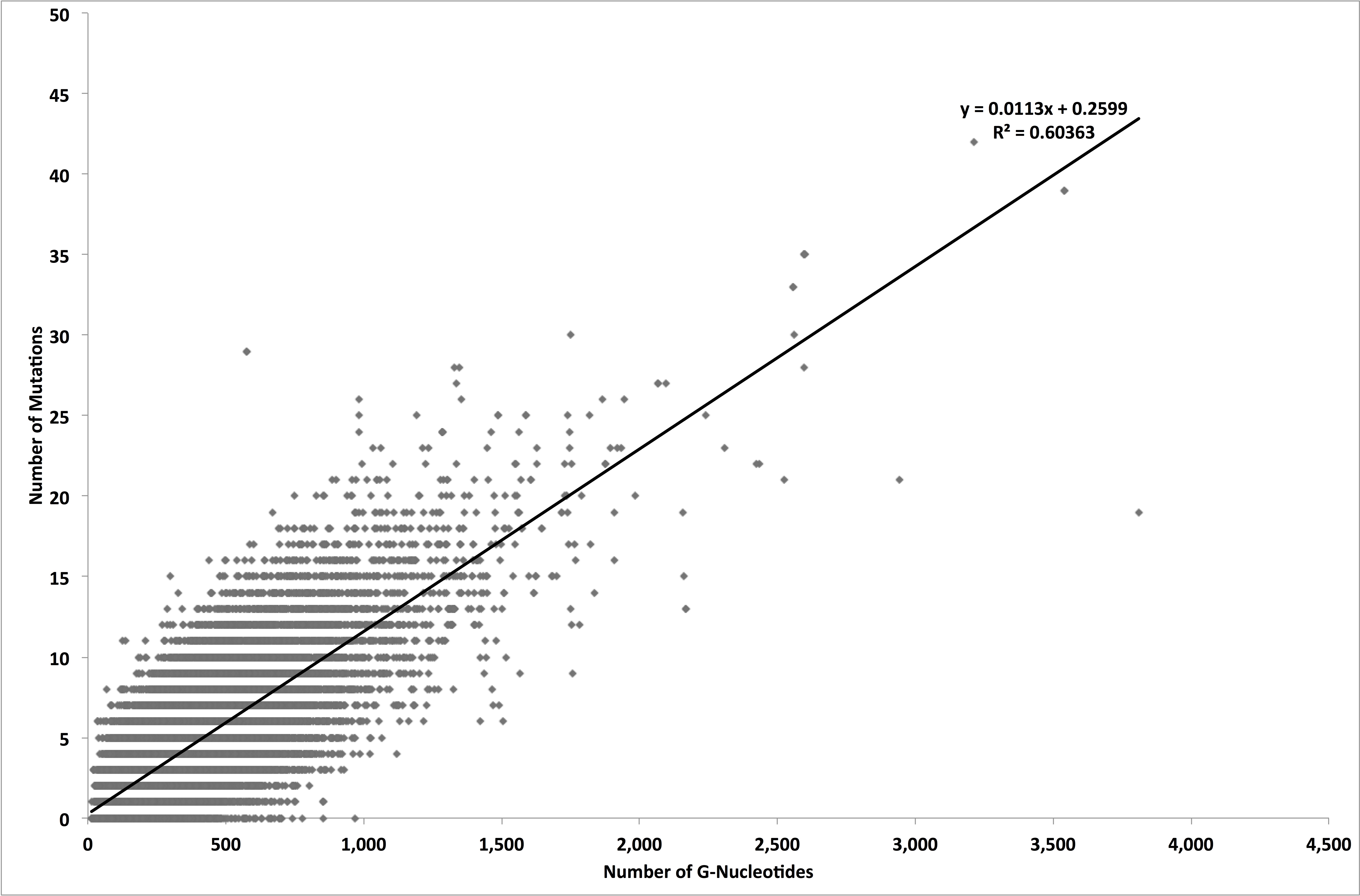

Supplement: Supplementary file 5 [file 1079FigureS5.jpg]

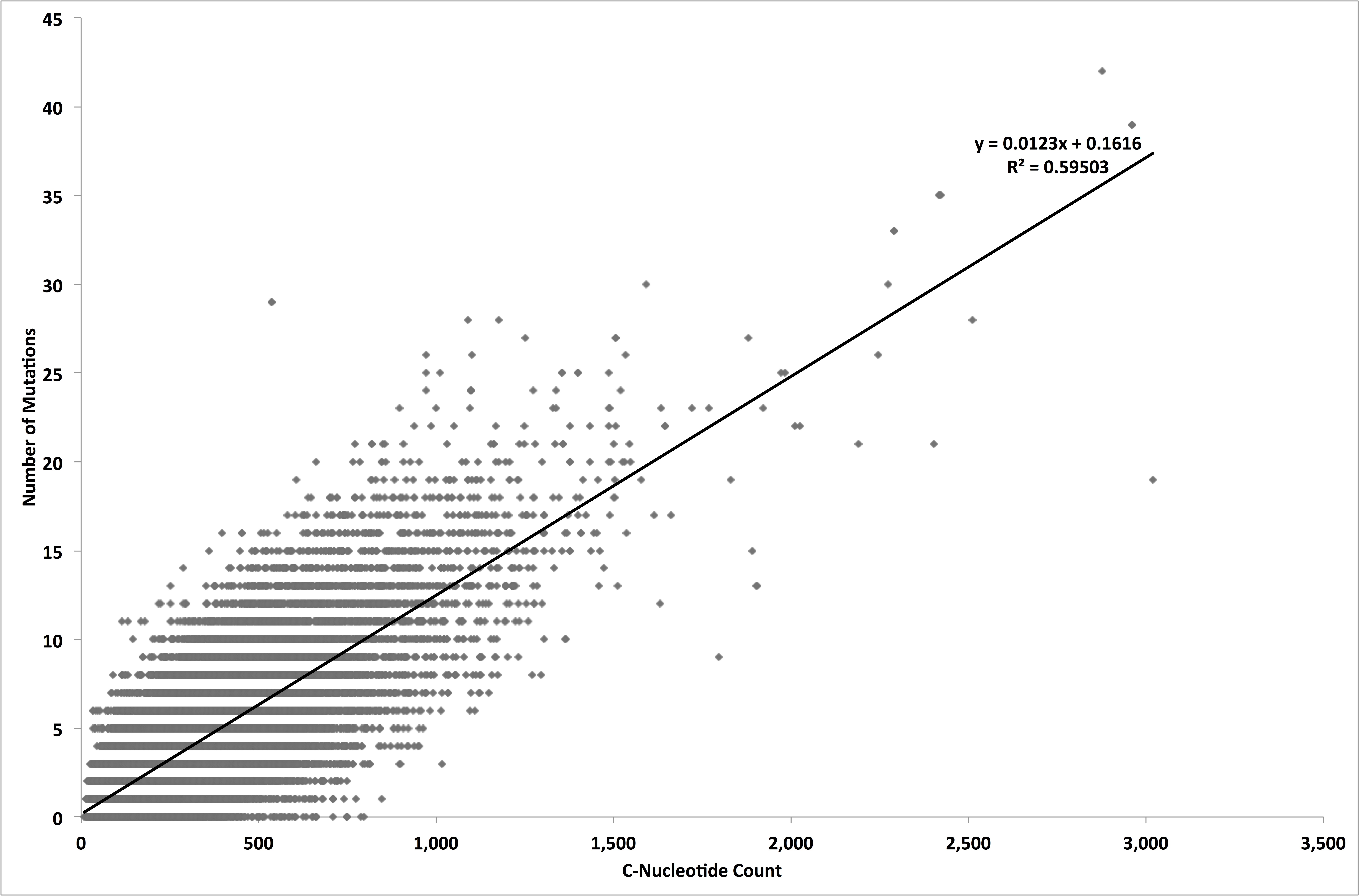

Supplement: Supplementary file 6 [file 1079FigureS6.jpg]

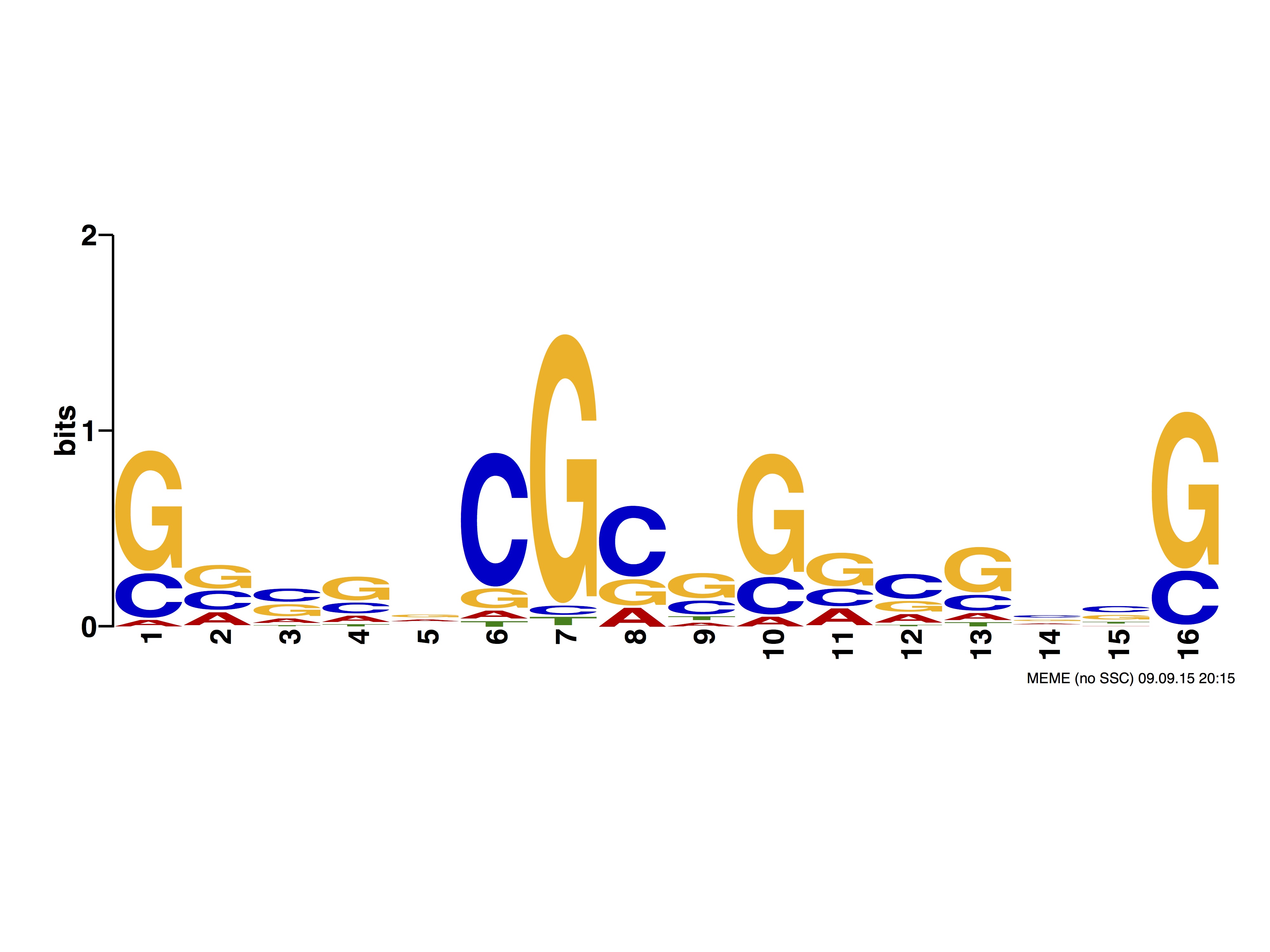

Supplement: Supplementary file 7 [file 1079FigureS7.jpg]

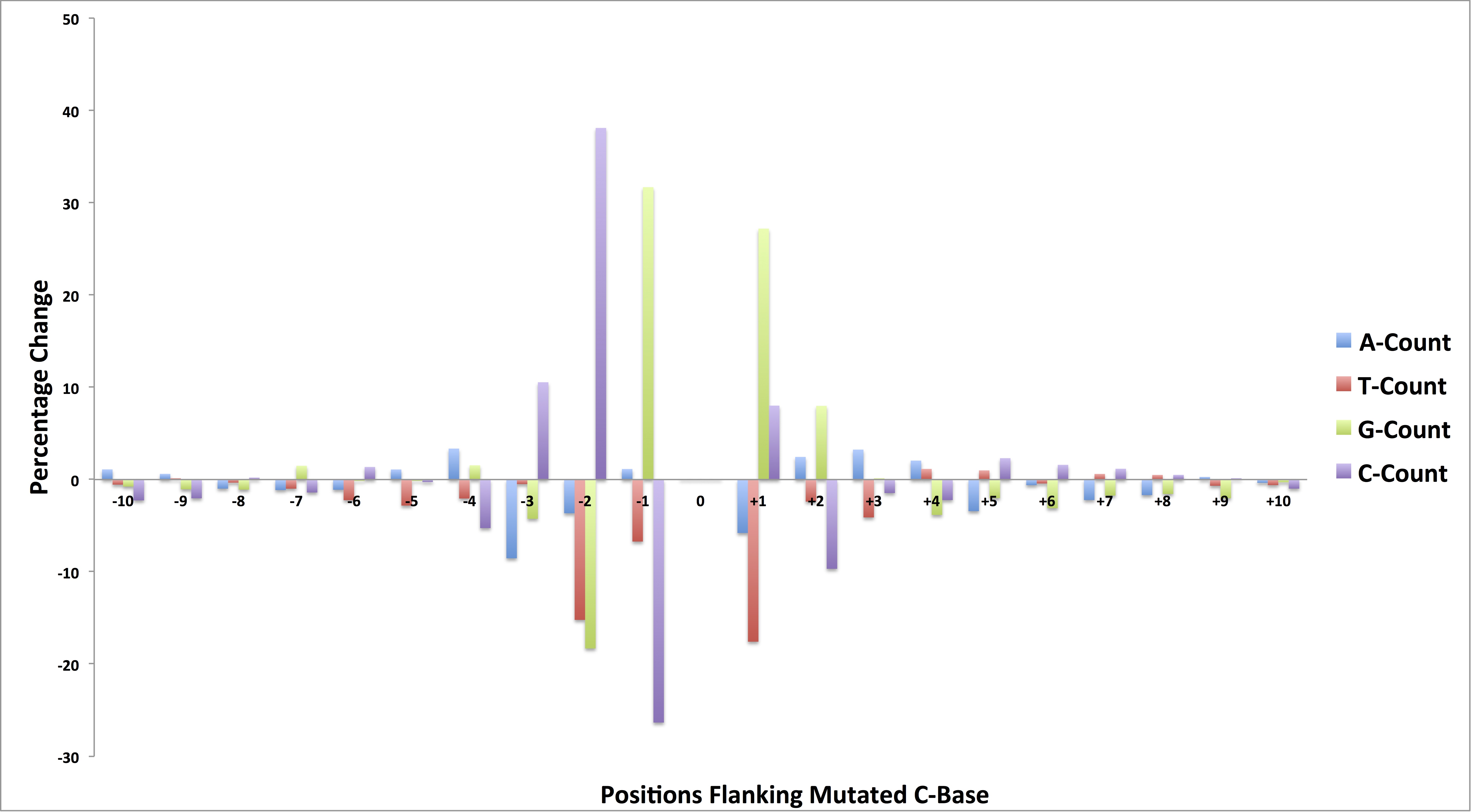

Supplement: Supplementary file 8 [file 1079FigureS8.jpg]

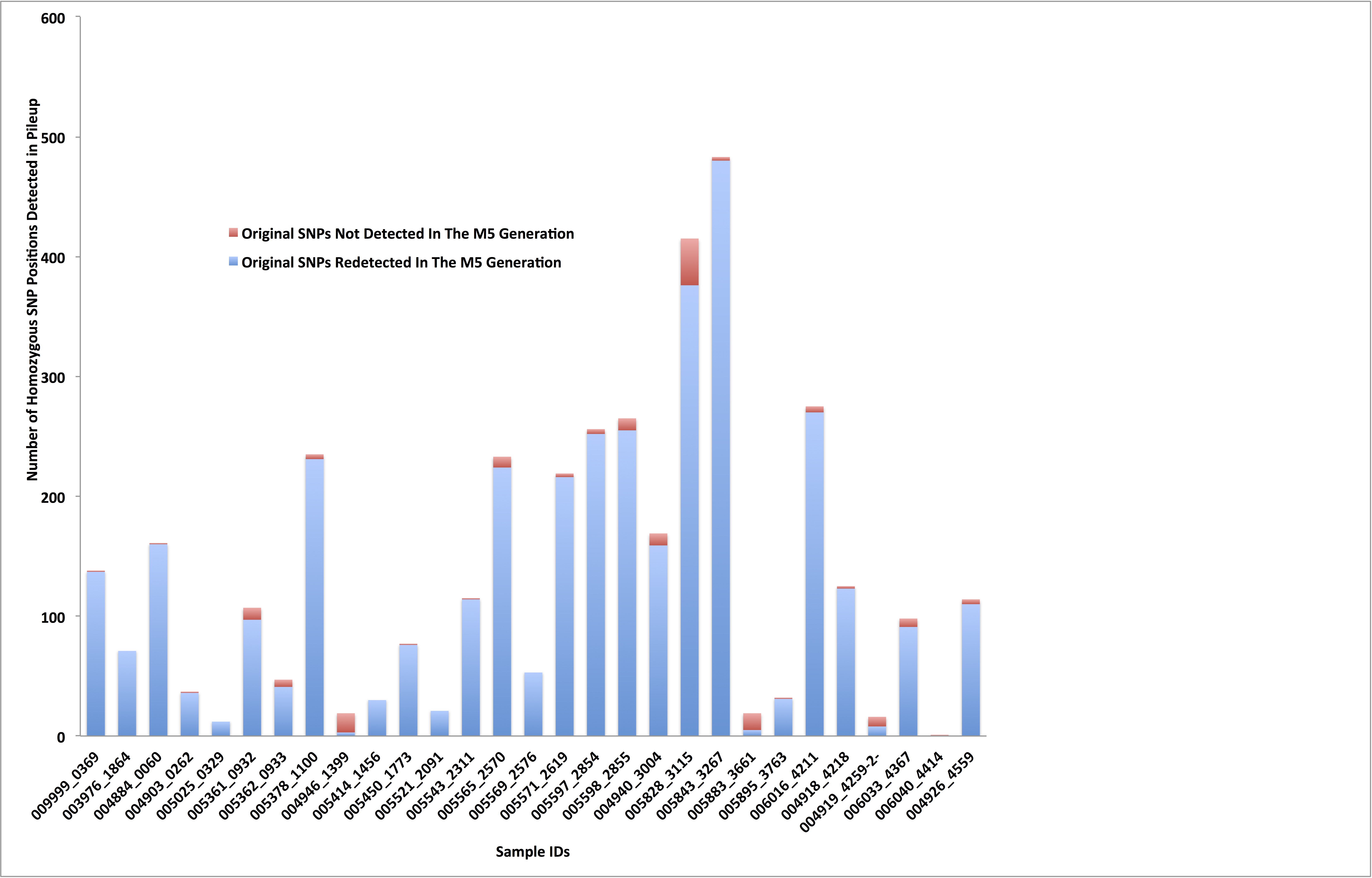

Supplement: Supplementary file 9 [file 1079FigureS9.jpg]

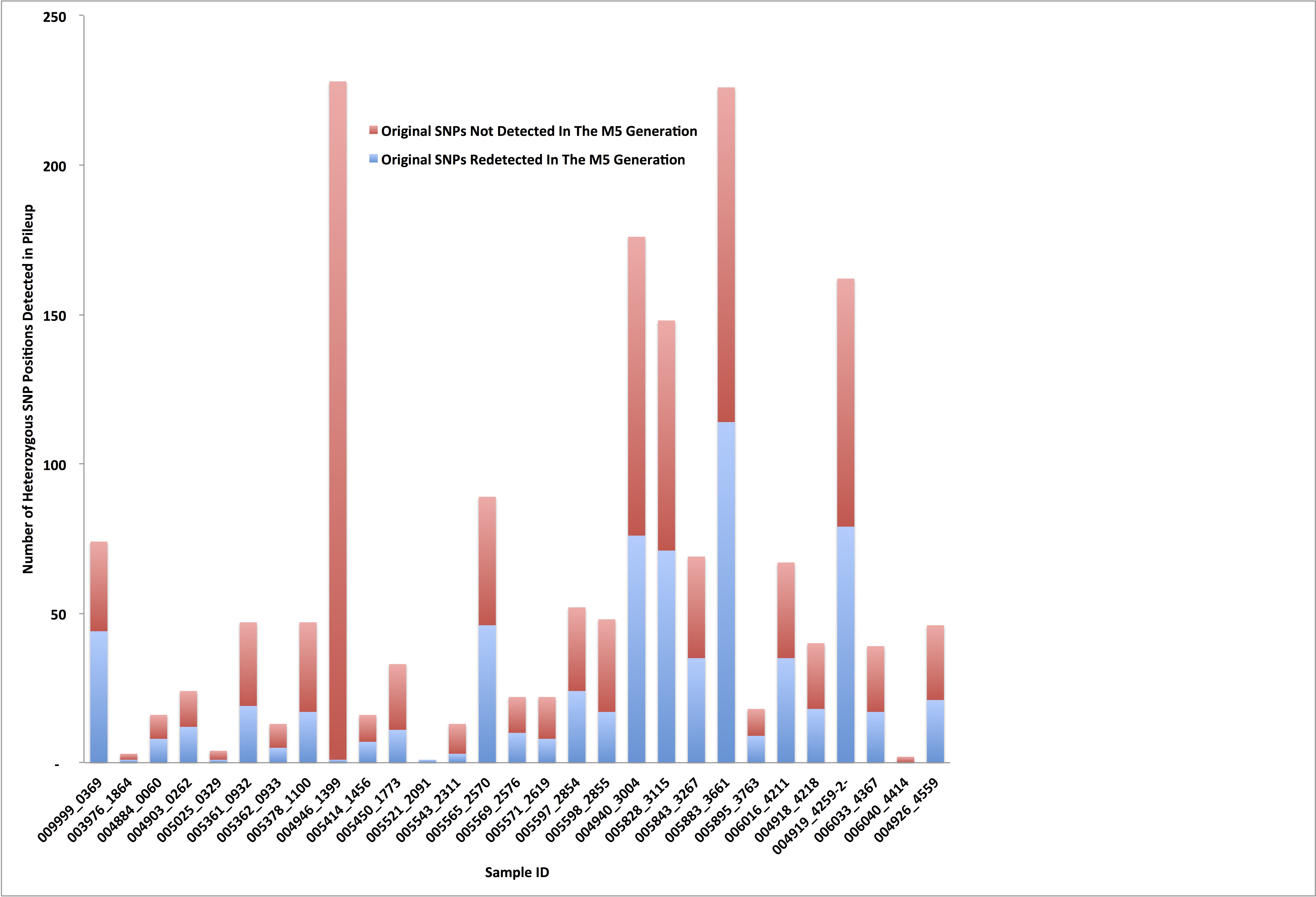

Supplement: Supplementary file 10 [file 1079FigureS10.jpg]
