# Supplementary material for: Whole-Genome Sequence Accuracy Is Improved by Replication in a Population of Mutagenized Sorghum
Source: G3 (Bethesda). 2018 Jan 25;8(3):1079–94. doi: 10.1534/g3.117.300301 (PMC5844295; doi:10.1534/g3.117.300301)
Supplement: Supplementary file 11 [file 1079FileS26.docx]

**Supplemental Table S1.** Frequency distribution of genomic positions for replicate SNPs predicted repeatedly in the 570 EMS-treated sorghum individuals.

| **Number of Individuals** | **Number of Genomic Positions** | **SNPs Count** |
| --- | --- | --- |
| 2-50 | 42,424 | 356,850 |
| 51-100 | 3,545 | 256,951 |
| 101-150 | 2,283 | 284,416 |
| 151-200 | 1,712 | 298,498 |
| 201-250 | 1,466 | 329,905 |
| 251-300 | 1,285 | 352,453 |
| 301-350 | 1,231 | 400,990 |
| 351-400 | 1,126 | 422,004 |
| 401-450 | 1,037 | 440,956 |
| 451-500 | 1,085 | 515,902 |
| 501-570 | 678 | 352,488 |
| **Total** | **57,872** | **4,011,413** |

**Supplemental Table S2.** Mutation spectrum of the standard filtered SNP calls in the 570 EMS-mutagenized sorghum BTx623 individuals.

|  | **Homozygous SNPs** | | | **Heterozygous SNPs** | | |
| --- | --- | --- | --- | --- | --- | --- |
|  | **All** | **Redundant** | **Unique** | **All** | **Redundant** | **Unique** |
| **A->G** | 589,986 | 585,456 | 4,530 | 1,027,490 | 1,022,616 | 4,874 |
| **A->T** | 222,831 | 219,766 | 3,065 | 340,402 | 337,464 | 2,938 |
| **A->C** | 208,555 | 207,313 | 1,242 | 269,942 | 268,967 | 975 |
| **G->A** | 1,027,845 | 424,322 | 603,523 | 1,272,951 | 1,057,127 | 215,824 |
| **G->T** | 289,557 | 286,635 | 2,922 | 287,019 | 284,052 | 2,967 |
| **G->C** | 268,467 | 266,427 | 2,040 | 386,398 | 384,396 | 2,002 |
| **T->A** | 228,101 | 224,972 | 3,129 | 370,442 | 367,375 | 3,067 |
| **T->G** | 220,384 | 219,170 | 1,214 | 266,145 | 265,107 | 1,038 |
| **T->C** | 594,495 | 589,959 | 4,536 | 1,022,416 | 1,017,636 | 4,780 |
| **C->A** | 328,243 | 325,332 | 2,911 | 298,493 | 295,461 | 3,032 |
| **C->G** | 243,391 | 241,357 | 2,034 | 380,364 | 378,290 | 2,074 |
| **C->T** | 1,022,321 | 420,704 | 601,617 | 1,315,553 | 1,095,963 | 219,590 |
| **Total** | **5,244,176** | **4,011,413** | **1,232,763** | **7,237,615** | **6,774,454** | **463,161** |

**Supplemental Table S3.** Mutation spectrum of the standard filtered SNP calls in the 586 EMS-mutagenized sorghum BTx623 individuals.

|  | **Homozygous SNPs** | | | **Heterozygous SNPs** | | |
| --- | --- | --- | --- | --- | --- | --- |
|  | **All** | **Redundant** | **Unique** | **All** | **Redundant** | **Unique** |
| **A->G** | 606,561 | 601,853 | 4,708 | 1,056,494 | 1,051,453 | 5,041 |
| **A->T** | 229,188 | 226,017 | 3,171 | 350,218 | 347,181 | 3,037 |
| **A->C** | 214,225 | 212,933 | 1,292 | 277,673 | 276,661 | 1,012 |
| **G->A** | 1,061,030 | 436,074 | 624,956 | 1,309,550 | 1,087,015 | 222,535 |
| **G->T** | 297,774 | 294,735 | 3,039 | 295,284 | 292,237 | 3,047 |
| **G->C** | 275,947 | 273,827 | 2,120 | 397,443 | 395,355 | 2,088 |
| **T->A** | 234,469 | 231,219 | 3,250 | 380,959 | 377,790 | 3,169 |
| **T->G** | 226,608 | 225,343 | 1,265 | 273,641 | 272,565 | 1,076 |
| **T->C** | 611,321 | 606,628 | 4,693 | 1,051,284 | 1,046,318 | 4,966 |
| **C->A** | 337,551 | 334,526 | 3,025 | 306,979 | 303,844 | 3,135 |
| **C->G** | 250,213 | 248,091 | 2,122 | 391,298 | 389,141 | 2,157 |
| **C->T** | 1,054,606 | 432,375 | 622,231 | 1,352,934 | 1,126,683 | 226,251 |
| **Total** | **5,399,493** | **4,123,621** | **1,275,872** | **7,443,757** | **6,966,243** | **477,514** |

**Supplemental Table S4.** The G:C to A:T percentages of standard filtered SNPs calculated for the SNP replicate categories in the 570 EMS-mutagenized sorghum BTx623 individuals.

| **Number of Replicates** | **G:C -> A:T (%)** | |
| --- | --- | --- |
|  | **Homozygous** | **Heterozygous** |
| **1** | 98 | 94 |
| **2** | 61 | 53 |
| **3** | 41 | 36 |
| **4** | 40 | 33 |
| **5** | 37 | 34 |
| **6** | 36 | 33 |
| **7** | 36 | 33 |
| **8** | 34 | 33 |
| **9** | 31 | 32 |
| **>=10** | 24 | 32 |

**Supplemental Table S5.** Classification of the predicted EMS-induced SNPs and their effects in the 570 EMS-mutagenized sorghum BTx623 individuals

| **SNPs Classification** | **Total** | | **Mean** | |
| --- | --- | --- | --- | --- |
|  | **Homozygous** | **Heterozygous** | **Homozygous** | **Heterozygous** |
| **Stop Gained** | 2,776 | 1,317 | 5 | 2 |
| **Splice Site Donor** | 588 | 298 | 1 | 1 |
| **Splice Site Acceptor** | 413 | 101 | 1 | 0 |
| **Start Lost** | 107 | 33 | 0 | 0 |
| **Stop Lost** | 1 | 3 | 0 | 0 |
| **High Impact** | 3,885 | 1,752 | 7 | 3 |
| **Moderate Impact** | 54,412 | 19,277 | 95 | 34 |
| **Start Gained** | 3,255 | 1,202 | 6 | 2 |
| **5' Prime UTR** | 15,714 | 5,212 | 28 | 9 |
| **3' Prime UTR** | 24,176 | 9,581 | 42 | 17 |
| **Introns** | 105,307 | 41,118 | 185 | 72 |
| **Intergenic** | 997,889 | 375,723 | 1,751 | 659 |

**Supplemental Table S6.** Distribution of synonymous stop codon lost substitutions in the 586 EMS-mutagenized sorghum BTx623 individuals.

|  | **Homozygous** | **Heterozygous** | **Total** |
| --- | --- | --- | --- |
| **TGA** | 64 | 28 | 92 |
| **TAG** | 50 | 6 | 56 |
| **TAA** | 0 | 0 | 0 |
| **Total** | 114 | 34 | 148 |

**Supplemental Table S7.** Summary statistics for the detection and annotation of indels in the 570 EMS-mutagenized sorghum BTx623 individuals.

|  | **Homozygous Indels** | **Heterozygous Indels** |
| --- | --- | --- |
| **Q20 Filtered** | 2,566,405 | 656,021 |
| **Standard Filtered** | 1,613,046 | 321,735 |
| **Non-Replicate** | 9,694 | 7,435 |
| **High Impact** | 121 | 145 |
| **Moderate Impact** | 71 | 99 |
| **Splice Site Acceptor** | 6 | 3 |
| **Splice Site Donor** | 9 | 4 |
| **5' Prime UTR** | 231 | 348 |
| **3' Prime UTR** | 270 | 210 |
| **Introns** | 975 | 898 |
| **Intergenic** | 8,060 | 5,778 |

**Supplemental Table S8.** Summary statistics for the detection and annotation of indels in the 586 EMS-mutagenized sorghum BTx623 individuals.

|  | **Homozygous Indels** | **Heterozygous Indels** |
| --- | --- | --- |
| **Q20 Filtered** | 2,638,564 | 674,227 |
| **Standard Filtered** | 1,658,457 | 330,621 |
| **Non-Replicates** | 10,074 | 7,686 |
| **High Impact** | 128 | 149 |
| **Moderate Impact** | 75 | 105 |
| **Splice Site Acceptor** | 6 | 3 |
| **Splice Site Donor** | 9 | 4 |
| **5' Prime UTR** | 245 | 358 |
| **3' Prime UTR** | 278 | 213 |
| **Introns** | 1,015 | 932 |
| **Intergenic** | 8,369 | 5,972 |

**Supplemental Figure S1.** The mutation spectrum of the standard filtered SNPs predicted for the sorghum EMS-mutagenized population. The majority (61% of the homozygous and 64% of the heterozygous) of the SNPs were not G:C to A:T transitions and hence not likely to be products of EMS mutagenesis.

**Supplemental Figure S2.** The mutation spectrum of the non-replicate homozygous and heterozygous SNPs detected in the 570 EMS-treated sorghum individuals. Removal of the replicate SNPs with origin in the error-prone genomic positions resulted in predominantly G:C to A:T substitutions. The non-replicate SNPs are most likely EMS-induced SNPs.

**Supplemental Figure S3.** The mutation spectrums at the sorghum genomic position categories for the homozygous standard-filtered SNPs. Categories were based on the count of the number of sorghum individuals having a SNP prediction at the genomic position. For the signal-to-noise analysis the percentage of G:C to A:T substitutions served as the signal intensity of bona fide EMS mutagenesis activity.

**Supplemental Figure S4.** The length distribution of the predicted EMS-induced insertion/deletion polymorphisms in the 586 EMS-mutagenized sorghum individuals. The deletions and insertions are depicted as negative and positive lengths respectively.

**Supplemental Figure S5.** The linear correlation between the predicted number of EMS-induced mutations and the total number of G-nucleotide residues in a gene transcript.

**Supplemental Figure S6.** The linear correlation between the predicted number of EMS-induced mutations and the total number of C-nucleotide residues in a gene transcript.

**Supplemental Figure S7.** Analysis of the 21nt sequence context of 10,000 randomly selected EMS-induced homozygous mutations in the 586 resequenced *sorghum bicolor* genomes. Using the MEME ungapped motif finder the topmost motif (p-value of 5.7e-304) is 16 bases long and is predominantly CG-rich. The predominant EMS-induced mutational target is the G-nucleotide at position 7.

**Supplemental Figure S8.** The 21nt sequence contexts comparison for the mutated C-residue DNA sites in the EMS-induced SNPs and randomly selected positions in the sorghum reference genome. The percentage change in the nucleotide frequency for all DNA base types in all DNA positions shows an overrepresentation of a 5’ -CG**C**G-3’ motif. The mutated C-residue is shown in bold.

**Supplemental Figure S9.** Confirmation of the EMS-induced homozygous SNP positions in the resequenced genomes of the M_5_ generation of 29 randomly selected subset of the original 586 EMS-mutagenized sorghum individuals.

**Supplemental Figure S10.** Confirmation of the EMS-induced heterozygous SNP positions in the resequenced genomes of the M_5_ generation of 29 randomly selected subset of the original 586 EMS-mutagenized sorghum individuals.

List of Supplemental Data Files:

1. Supplemental File S1: Standard filtered SNPs detected in each of the 586 sorghum individuals.

2. Supplemental File S2: Probable error-prone SNP genomic positions in the sorghum reference genome (version 2.1).

3. Supplemental File S3: Non-replicate, and likely EMS-induced homozygous SNPs in each of the 586 sorghum individuals.

4. Supplemental File S4: Non-replicate, and likely EMS-induced heterozygous SNPs in each of the 586 sorghum individuals.

5. Supplemental File S5: Counts of homozygous and heterozygous SNPs in all the 586 sequenced lines.

6. Supplemental File S6: Replicate, and likely false-negative EMS-induced homozygous G:C to A:T SNPs in each of the 586 sorghum individuals.

7. Supplemental File S7: Replicate, and likely false-negative EMS-induced heterozygous G:C to A:T SNPs in each of the 586 sorghum individuals.

8. Supplemental File S8: SnpEff functional classification of the homozygous EMS-induced SNPs in all the 586 sorghum individuals.

9. Supplemental File S9: Function description for the genes containing the SnpEff-annotated homozygous EMS-induced SNPs in all the 586 sorghum individuals.

10. Supplemental File S10: List of SnpEff-annotated EMS-induced SNPs predicted to trigger silent stop codon lost substitutions in genes of the 586 sorghum individuals.

11. Supplemental File S11: SnpEff functional classification of the heterozygous EMS-induced SNPs in all the 586 sorghum individuals.

12. Supplemental File S12: Function description for the genes containing the SnpEff-annotated heterozygous EMS-induced SNPs in all the 586 sorghum individuals.

13. Supplemental File S13: Function description for the genes containing the SnpEff-annotated tentative false-negative homozygous EMS-induced SNPs in all the 586 sorghum individuals.

14. Supplemental File S14: Function description for the genes containing the SnpEff-annotated tentative false-negative heterozygous EMS-induced SNPs in all the 586 sorghum individuals.

15. Supplemental File S15: SnpEff functional classification of the likely EMS-induced indels in all the 586 sorghum individuals.

16. Supplemental File S16: Function description for the genes containing the SnpEff-annotated likely EMS-induced indels in all the 586 sorghum individuals.

17. Supplemental File S17: SIFT prediction results for the missense-annotated homozygous EMS-induced SNPs in all the 586 sequenced individuals.

18. Supplemental File S18: SIFT prediction results for the missense-annotated heterozygous EMS-induced SNPs in all the 586 sequenced individuals.

19. Supplemental File S19: Summary statistics for the sequencing, mapping, variants prediction, filtering, annotation and classification for the detected EMS-induced variants in all 586 sequenced individuals.

20. Supplemental File S20: Detailed classification, gene function description and annotation of the medium or high impact homozygous EMS-induced SNPs.

21. Supplemental File S21: Detailed classification, gene function description and annotation of the medium or high impact heterozygous EMS-induced SNPs.

22. Supplemental File S22: Detailed classification, gene function description and annotation of the medium or high impact EMS-induced indels.

23. Supplemental File S23: The detailed attributes of gene transcripts, including number of SNPs, GC-content, sequence length, mononucleotide and dinucleotide counts, which were used in the linear correlation analysis.

24. Supplemental File S24: The subset of BLAST ungapped global alignments for the 51nt sequence contexts of the error-prone SNP positions, randomly selected EMS-induced SNP positions and randomly selected genome positions.

25. Supplemental File S25: Sample scripts for the variants detection and annotation pipeline.
